# A Dynamically Consistent Nonstandard Difference Scheme for a Discrete-Time Immunogenic Tumors Model

**DOI:** 10.3390/e24070949

**Published:** 2022-07-07

**Authors:** Muhammad Salman Khan, Maria Samreen, Muhammad Asif Khan, Manuel De la Sen

**Affiliations:** 1 Department of Mathematics, Quaid-I-Azam University, Islamabad 44230, Pakistan; mskhan@math.qau.edu.pk; 2 Department of Mathematics, Kahota-Haveli Campus, University of the Poonch Rawalakot, Rawalakot 12350, Pakistan; asif31182@gmail.com; 3 Department of Electricity and Electronics, Institute of Research and Development of Processes, Faculty of Science and Technology, University of the Basque Country (UPV/EHU), Campus of Leioa, 48940 Leioa, Bizkaia, Spain; manuel.delasen@ehu.eus

**Keywords:** immunogenic tumors model, nonstandard difference scheme, Andronov–Hopf bifurcation, boundedness, existence, linearized stability, Neimark–Sacker bifurcation, control of bifurcation, numerical simulations

## Abstract

This manuscript deals with the qualitative study of certain properties of an immunogenic tumors model. Mainly, we obtain a dynamically consistent discrete-time immunogenic tumors model using a nonstandard difference scheme. The existence of fixed points and their stability are discussed. It is shown that a continuous system experiences Hopf bifurcation at one and only one positive fixed point, whereas its discrete-time counterpart experiences Neimark–Sacker bifurcation at one and only one positive fixed point. It is shown that there is no chance of period-doubling bifurcation in our discrete-time system. Additionally, numerical simulations are carried out in support of our theoretical discussion.

## 1. Introduction

A tumor is a cluster of infections produced by the irregular evolution of cells described by DNA latent to a blowout in additional body parts. In every category of a tumor, certain cells in the body develop strangely and damage the nearby tissues. The healthy physique has a strict immune arrangement to protect it in case of tumors caused by the letdown of the immune system and the supplementary mechanism inside the body [1]. Tumor cases have been increasing very rapidly in recent eras, and it has been estimated that approximately 18.1 million persons suffer from them each year, out of which 9.6 million die [2]. The incidence of new tumors has been ascending, and it is projected that the number may reach 22.2 million by 2030 [3]. Therefore, it is necessary to improve novel, progressive, and cost-effective approaches to deal with these circumstances. Presently, the most public tumor treatments are chemotherapy [4], immunotherapy ([5,6]), surgery [7], and radiotherapy [8]. Despite all of these management options, it reverts. Thus it is essential for additional and operational treatment to be understandable. The immune reaction to a tumor is frequently cell-refereed cytotoxic T lymphocytes (*CTL*), and natural killer (*NK*) cells show a leading character. Various scientific models of the interactions between the increasing tumor and immune system have been established [9,10,11,12]. Moreover, several mathematical models describe the kinetics of cells refereed cytotoxicity in vitro [13,14,15]. With these mathematical models, several occurrences understood, statistical estimates for biologically essential factors have been acquired, and guesses made. The qualitative analysis of anti-tumor immune response in vivo is complicated and not well-understood. Freely ascending cancers have little immunogenicity, and frequently spread out of control in most creatures. Sometimes, the escape of any tumor from immune reconnaissance is connected with many different applications, namely, the damage or covering of cancer antigens, cancer-influenced disorders in safe regulation, damage to *MHC* class-I particles, and the addition of a variety of cancer duplicates resistant to cytolytic mechanisms [16,17,18]. Although attacking cells of the immune system may kill tumor cells, protected reconnaissance of natural cancers may be operative and significant in keeping tumor frequency low. The leading efforts to improve arrangements for immunotherapy or its grouping with other treatment approaches focus on reducing cancer mass. However, the bulk of such efforts remain ineffective. The critical explanation for this is that even after “effective” and “clinically comprehensive” elimination of cancer cells, a small number of “remaining” cancer cells remain, which may develop into subordinate cancers or “latent” metastases.

Cancer dormancy is a functioning span used to define a state in which a possibly dead cancer cells persevere for a protracted time with slight or no growth in the cancer cell population. It is commonly supposed that cancer cells do not develop at a speedy frequency throughout the dormancy period, apparently due to the nonappearance of a factor required for advanced evolution into cancer [19,20,21]. Nevertheless, a substitute probability is that quickly increasing cells are exterminated at a frequency equivalent to their creation. Undeveloped conditions develop both during essential treatment for cancer, and in the initial phases of cancer development. In truth, there is a typical arrangement in that neoplastic cells discharge from significant cancer very initial in its growth in any person [22]. The providence of these evading neoplastic compartments regulates whether the enduring cancer survives or kills the tumor. The straight contribution of *CTL* in the provision of a latent cancer state has been revealed in certain specific investigational models. Moreover, *CTL*’s different kinds of protected system cells, such as *NK* cells and macrophages, may preserve a latent cancer state [22].

In the past two decades, many authors have presented various clarifications for the expiry of a latent cancer state, snitching over cancers, and immune inspiration properties. Frequently, these clarifications are centred on the concepts of protected range, antigenic variation, creation by cancer cells of unlike kinds of immune cell delaying factors, group of immune suppressor cells, variations in auto-governing systems in a cancer localization area, and other new complex concepts that are very challenging to verify or invalidate experimentally. We consider that these occurrences might result from nonlinear dynamic connections between cancer and the insusceptible system [23,24,25,26]. The authors of [26] have examined a simple scientific model of a compartment-refereed reaction to a developing cancer compartment population. They have explained that their model contrasts with most models in the literature because it explains the penetration of cancer by consequence or cells and the probability of consequence or cell in the beginning. Kuznetsov [26] have studied an alternative to this model. Moreover, ref. [26] discussed the qualitative conduct of the structure using methods from the bifurcation concept. They applied the model to discuss the appliances of cancer latency and snitching through. They discussed that a non-zero frequency of consequence or cell, in the beginning, is necessary to achieve snitching through. They have found that snitching over, cancer latency, and the immune motivation of cancer development, properties which have been investigated independently, might be connected, which is similar to our model. Here, we study cancers with cells that are “immune genetic”, and consequently focus on the insusceptible attack by cytotoxic effector cells, for example, *CTL*, or *NK* cells. The communication among effector cells *(EC)* and cancer cells (*TC*) in vitro can defined using the kinetic scheme below.

**Figure 1:**
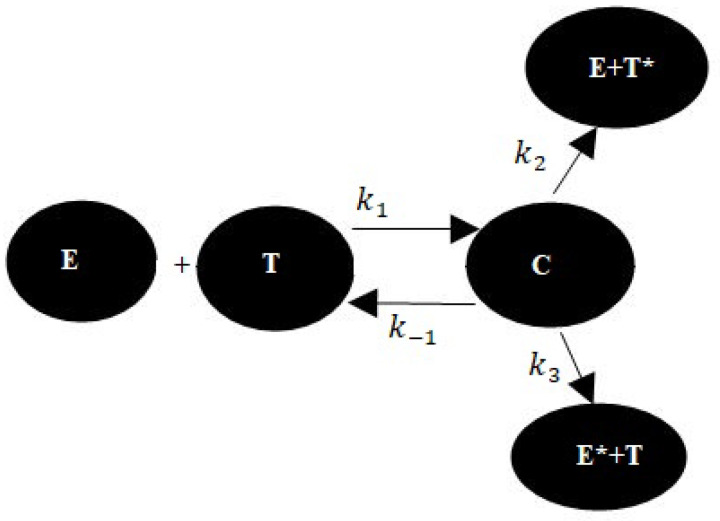
Data flow diagram for our system.

Where, in Figure 1, $T,E,C,T^{*},E^{*}$ are the limited meditations of cancer cells, effector cells, effector cell–cancer cell conjugates, “critically hit” *TC* cells and deactivated effector cells, respectively. Critically hit cancer cells are intended to die. They similarly have been named cells “encoded to die”. The addition of deactivated effector cells is a strange feature of this model. In cases with a lesser degree of *CTL*, culture appears to have a limited ability to constantly destroy target cells [27]. This is because particle collapse is responsible for cytotoxic influences or other controlling effects, probably due to the discharge of particles from the cancer cell after *EC* and *TC* are conjugated. Moreover, $k_{1}$, $k_{-1}$, $k_{2}$, and $k_{3}$ are non-negative kinetic real numbers; $k_{1}$ and $k_{-1}$ refer to the degrees of binding of *TC* from *EC* and objectivity of *TC* to *EC* without injuring cells, $k_{2}$ is the degree at which *EC* to *TC* connections conclusively program *TC* for lysis, and $k_{3}$ is the degree at which *EC* to *TC* connections deactivate *EC*. Hence, we have the following system of differential equations as a model for the communication among *EC* and increasing immunogenic cancer in vivo [27]:

(1) $$\left\{\begin{array}{c} \frac{dE}{dt}=s+G\left(C,T\right)-d_{1}E-k_{1}ET+\left(k_{-1}+k_{2}\right)C,\\ \frac{dT}{dt}=aT\left(1-bT\right)-k_{1}ET+\left(k_{-1}+k_{3}\right)C,\\ \frac{dC}{dt}=k_{1}ET-\left(k_{-1}+k_{2}+k_{3}\right)C,\\ \frac{dE^{*}}{dt}=k_{3}C-d_{2}E^{*},\\ \frac{dT^{*}}{dt}=k_{2}C+d_{3}T^{*}, \end{array}\right.$$

where

$$G\left(C,T\right)=\frac{fC}{g+T}.$$

The authors of [28] have explained that the last two equations from the system (1) are “slaves” to the first three equations in that system, as variables $T^{*}$ and $E^{*}$ have no influence on each other or the other variables in the system. Hence, in their work, they reduced the system (1) to the first three equations (see [28]) in order to dictate the dynamics of the system (1). Moreover, the development and division of cellular conjugates *C* follows the time measure of numerous tens of minutes to a limited number of hours. A time interval of this type is similarly detected before the disintegration of lethally hit tumor cells by separating the cell wall or membrane. However, the growth along with the inflow of effector cells into the spleen arises on a considerably slower time measure, possibly tens of hours. This inspires the application of a quasi-steady state estimate to the third equation of system (1) (that is, $\frac{dC}{dt}\approx 0$), which yields the following system of equations (see [28]):

(2) $$\left\{\begin{array}{c} \frac{dE}{dt}=s+\frac{pET}{g+T}-mET-dE,\\ \frac{dT}{dt}=aT\left(1-bT\right)-nET, \end{array}\right.$$

where $p=fK,\;m=Kk_{3},\;n=Kk_{2},$ and $d=d_{l}$ are dimensional parameters. In addition, for better study of the dynamics of model (2) it is necessary to non-dimensionalize the system (2).

### Non-Dimensionalization of System

The non-dimensionalized form of model (2) is obtained by selecting an order of degree application measure for *E* and *T* cell populations, $E_{0}$ and $T_{0}$, respectively, as proposed from the tests discussed in the previous section: $E_{0}$ = $T_{0}$ = $10^{6}$ cells (see [28]). Time is scaled comparative to the degree of cancer cell deactivation, that is, $\tau =nT_{0}t.$ Formally, the model can be re-articulated as

(3) $$\left\{\begin{array}{c} \frac{dx}{dt}=\sigma +\frac{\rho x(t)y(t)}{\eta +y(t)}-\mu x\left(t\right)y\left(t\right)-\delta x\left(t\right),\\ \frac{dy}{dt}=\alpha y\left(t\right)\left(1-\beta y\left(t\right)\right)-x\left(t\right)y\left(t\right), \end{array}\right.$$

where $x=\frac{E}{E_{0}},\;\;y=\frac{T}{T_{0}}$ and parameter estimates for system (3) are the following (see Table 1):

The authors of [29] considered the post-collapsing conduct of functionally classified curved shell sections. Moreover, they investigated different shell geometries (cylindrical, elliptical, spherical, and hyperbolic) under the biaxial and uniaxial edge density. Duan et al. [30] have discussed a chemotherapy system’s stability analysis in cancer–immune responses. Moreover, their system has more than one fixed point. In order to conduct a stability analysis of these fixed points, they computed the upper Lyapunov exponents of the linearized system for these fixed points. They show that, while one fixed point is globally asymptotically stable if the noise is weak, another fixed point is constantly unstable whether the noise is weak or strong. We refer readers to [31] for further information on cancer–immune response systems. The authors of [32] discussed the discrete-time counterpart of a tumor–immune interaction model and analyzed the bifurcation in that model in fractional form. The authors of [33] studied the prime control for a tumor behaviour mathematical model using Atangana–Baleanu–Caputo fractional derivatives. In [34], the modeling and study of the dynamics of cancer virotherapy with an immune response were considered. For further study of attractive models related to tumor dynamics, we refer the reader to [35,36,37]. It is suitable to analyze any biological system’s qualitative behaviour by discrete-time systems compared to their alternatives in differential equations. Additionally, there is superior observation and investigation of chaos in all biological systems through discrete-time mathematical systems [38]. Hence, it is motivating to study the qualitative behaviour of the system (3) in its discrete form. In recent times, numerous scientific approaches have been presented to discretize any scientific model from continuous time. The traditional method is to use ordinary difference systems such as Euler’s approximations and Runge–Kutta methods to attain this objective.

Nevertheless, mathematical unpredictability is experienced using traditional finite difference approaches. To escape from this mathematical unpredictability it is possible to use a nonstandard finite difference technique, as specified by Mickens [39]. Generally, a nonstandard finite difference scheme is directed to protect the following characteristics of the corresponding continuous-time system: boundedness, the positivity of results, stability of fixed points, and bifurcations. The development of these varieties of difference methods is not straightforward, and no usual methods can be found in the literature for building them, possibly reflecting a chief disadvantage of nonstandard difference methods. Therefore, by applying a Mickens-type nonstandard scheme to model (3), we obtain the following discrete-time mathematical model (see [39,40]):

(4) $$\left\{\begin{array}{c} \frac{x_{n+1}-x_{n}}{h}=\sigma +\frac{\rho x_{n}y_{n}}{\eta +y_{n}}-\mu x_{n}y_{n}-\delta x_{n},\\ \frac{y_{n+1}-y_{n}}{h}=\alpha y_{n}\left(1-\beta y_{n}\right)-x_{n}y_{n} \end{array}\right.$$

where h∈[0,1) is the step size for discretization. Furthermore, system (4) can be transformed into the following form:

(5) $$\left\{\begin{array}{c} x_{n+1}=\frac{x_{n}+h\left(\sigma +\frac{\rho x_{n}y_{n}}{\eta +y_{n}}\right)}{1+h\left(\delta +\mu y_{n}\right)},\\ y_{n+1}=\frac{y_{n}\left(1+h\alpha \right)}{1+h\left(x_{n}+\alpha \beta y_{n}\right)}. \end{array}\right.$$

The subsequent parts of this manuscript are directed at: Andronov–Hopf bifurcation in system (3); the boundedness of solutions of system (5); the existence of fixed points and local stability analysis of system (5); the presence and direction of Neimark–Sacker bifurcation about the positive fixed point of system (5); the control of Neimark–Sacker bifurcation in system (5); and finally, several numerical simulations which support our theoretical discussion.

## 2. Andronov–Hopf Bifurcation

Let $\left(x^{*},y^{*}\right)$ be the positive fixed point of map (3). First, we explore the behavior of continuous system (3). In order to explore the behavior of system (3), the Jacobian matrix V(E) at $\left(x^{*},y^{*}\right)$ is provided by

$$V\left(E\right)=\begin{array}{c} \left[\begin{array}{cc} -\frac{\sigma }{x^{*}} & x^{*}\left(\frac{\eta \rho }{{\left(y^{*}+\eta \right)}^{2}}-\mu \right)\\ -y^{*} & \alpha -x^{*}-2y^{*}\alpha \beta \end{array}\right]. \end{array}$$

where $detV\left(E\right)=x^{*}y^{*}\left(\frac{\eta \rho }{{\left(y^{*}+\eta \right)}^{2}}-\mu \right)+\sigma +\frac{\alpha (2\beta y^{*}-1)\sigma }{x^{*}}$ and $TrV\left(E\right)=\alpha -2\alpha \beta y^{*}-\frac{\sigma }{x^{*}}-x^{*}.$ Hence, per the Routh–Hurwitz stability criterion, $\left(x^{*},y^{*}\right)$ is sink if and only if $x^{*}y^{*}\left(\frac{\eta \rho }{{\left(y^{*}+\eta \right)}^{2}}-\mu \right)+\sigma >\frac{\alpha \left(1-2\beta y^{*}\right)\sigma }{x^{*}}$ and $\alpha \left(1-2\beta y^{*}\right)<x^{*}+\frac{\sigma }{x^{*}}$ and source if and only if $x^{*}y^{*}\left(\frac{\eta \rho }{{\left(y^{*}+\eta \right)}^{2}}-\mu \right)+\sigma >\frac{\alpha \left(1-2\beta y^{*}\right)\sigma }{x^{*}}$ and $\alpha \left(1-2\beta y^{*}\right)>x^{*}+\frac{\sigma }{x^{*}}$. Moreover, system (3) experiences Hopf bifurcation if the parameters lie in the following curve:

$$T_{HB}=\left\{\left(\alpha ,\beta ,\delta ,\sigma ,\rho ,\mu ,\eta \right)\in R_{+}^{7}:\sigma =x^{*}\left(\alpha -x^{*}-2y^{*}\alpha \beta \right)\right\}.$$

**Figure 2:**
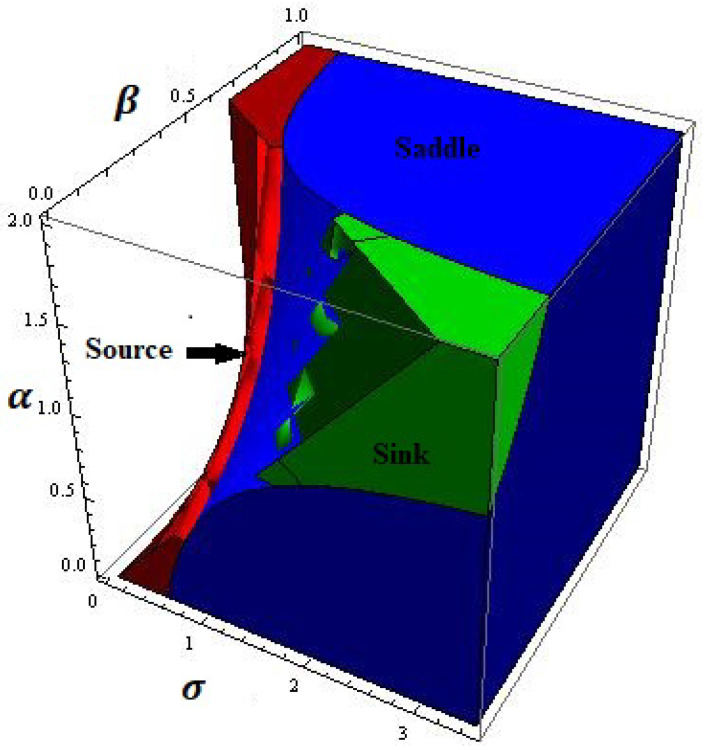
Topological classification of the one and only fixed point of system (3) for 0<α<2,0<β<1,δ=0.3743,η=20.19,μ=0.00311,ρ=11.131, and 0<σ<3.5 with initial conditions $x_{0}=1.6197$ and $y_{0}=0.82317$.

Furthermore, Figure 2 shows the topological classification of the fixed point $\left(x^{*},y^{*}\right)$. To explore the periodic nature of solutions of system (3), we study the exitances of subcritical and supercritical Hopf bifurcation. For this, we assume the next planar system:

(6) $$\begin{array}{c} \begin{array}{cc} \frac{dx}{dt}= & f(a,x,y),\\ \frac{dy}{dt}= & g(a,x,y), \end{array} \end{array}$$

where a∈R is the bifurcation parameter. Let $V(x^{*},y^{*})$ be the Jacobian matrix of (6) computed at equilibrium point $\left(x^{*},y^{*}\right)$. Moreover, the eigenvalues of (6) computed at any equilibrium point $\left(x^{*},y^{*}\right)$ are of the following form:

$$\lambda_{1,2}\left(a\right)=\phi \left(a\right)\pm \iota \varphi \left(a\right).$$

Furthermore, we assume that there is a particular value of the bifurcation parameter *a*, say $a_{0}$, for which the following conditions hold true (see [40]):

 **(i)**  $\phi (a_{0})=0$ and $\varphi \left(a_{0}\right)=\varphi_{0}\neq 0$. Then, there exists a conjugate pair of complex eigenvalues of $V(x^{*},y^{*})$ in the condition of non-hyperbolicity.
 **(ii)**  $\frac{d\phi (a)}{da}{|}_{a=a_{0}}=T\neq 0$, which is known as a transversality condition, that is, the eigenvalues of $V(x^{*},y^{*})$ cross the imaginary axis with non-zero speed [40].
 **(iii)**  There exists a discriminatory quantity $L(a_{0})\neq 0$, which is known as the first Lyapunov exponent (FLE) and is defined as follows:

$$L\left(a_{0}\right)=L_{1}+L_{2},$$

where

$$\left\{\begin{array}{c} L_{1}=\frac{1}{16}\left[f_{xxx}+f_{xyy}+g_{xxy}+g_{yyy}\right]\\ L_{2}=\frac{1}{16\varphi_{0}}\left[f_{xy}\left(f_{xx}+f_{yy}\right)-g_{xy}\left(g_{xx}+g_{yy}\right)-f_{xx}g_{xx}+f_{yy}g_{yy}\right] \end{array}\right.$$

with $f_{xy}=\frac{\partial^{2}f\left(a,x,y\right)}{\partial x\partial y}$ computed at $\left(x,y\right)=\left(x^{*},y^{*}\right)$ and $a=a_{0}.$

**Theorem** **1** ([40]). *Assume that conditions (i), (ii), and (iii) are satisfied; then, there exists a unique curve of periodic solutions. Moreover, this curve bifurcates from the fixed point into the region with $a>a_{0}$ if $L(a_{0})<0$ or $L(a_{0})>0$ if $a<a_{0}.$ In addition, the fixed point is stable whenever $a>a_{0}$ (respectively, $a<a_{0}$) and unstable for $a<a_{0}$ (respectively, $a>a_{0}$) for T<0 and T>0, respectively.*

It can be seen that periodic solutions are stable (respectively, unstable) if the fixed point is unstable (respectively, stable) on the side of $a=a_{0}$ where the periodic solutions exist. Keeping in view the above discussion for Andronov–Hopf bifurcation, we consider the system (3). For this, we assume that $4\left(\sigma +\frac{\alpha \sigma \left(2\beta y^{*}-1\right)}{x^{*}}+x^{*}y^{*}\left(\frac{\eta \rho }{{\left(\eta +y^{*}\right)}^{2}}-\mu \right)\right)-{\left(\frac{\sigma }{x^{*}}+x^{*}+\alpha \left(2\beta y^{*}-1\right)\right)}^{2}>0$; then, it is easy to see that the eigenvalues of the Jacobian matrix $V(x^{*},y^{*})$ are of the form

$$\lambda_{1,2}\left(\sigma \right)=\phi \left(\sigma \right)\pm \iota \varphi \left(\sigma \right),$$

$$\phi \left(\sigma \right)=\frac{1}{2}\left(\alpha -\frac{\sigma }{x^{*}}-x^{*}-2\alpha \beta y^{*}\right)$$

and

$$\varphi \left(\sigma \right)=\frac{1}{2}\sqrt{4\left(\sigma +\frac{\alpha \sigma \left(2\beta y^{*}-1\right)}{x^{*}}+x^{*}y^{*}\left(\frac{\eta \rho }{{\left(\eta +y^{*}\right)}^{2}}-\mu \right)\right)-{\left(\frac{\sigma }{x^{*}}+x^{*}+\alpha \left(2\beta y^{*}-1\right)\right)}^{2}}.$$

Next, ϕ(σ)=0 provides $\sigma =\sigma_{0}=x^{*}\left(\alpha -x^{*}-2\alpha \beta y^{*}\right)$. At $\sigma =\sigma_{0}$, we have

$$\varphi \left(\sigma_{0}\right)=\varphi_{0}=\sqrt{x^{*}\left(2\alpha +y^{*}\left(\frac{\eta \rho }{{\left(\eta +y^{*}\right)}^{2}}-4\alpha \beta -\mu \right)\right)-{\left(x^{*}\right)}^{2}-\alpha^{2}{\left(1-2\beta y^{*}\right)}^{2}}\neq 0.$$

For the transversality condition, we can see that

$${\frac{d\phi (\sigma )}{d\sigma }|}_{\sigma =\sigma_{0}}=-\frac{1}{2x^{*}}<0.$$

In order to shift the fixed point of system (3) to origin (0,0), we consider the following translations:

$$u\left(t\right)=x\left(t\right)-x^{*},v\left(t\right)=y\left(t\right)-y^{*}.$$

Moreover, by implementing this transformation on system (3), we obtain

(7) $$\begin{array}{c} \begin{array}{cc} \frac{du}{dt}= & \sigma +\frac{\rho \left(u+x^{*}\right)\left(v+y^{*}\right)}{\eta +y}-\mu \left(u+x^{*}\right)\left(v+y^{*}\right)-\delta \left(u+x^{*}\right),\\ \frac{dv}{dt}= & \alpha \left(v+y^{*}\right)\left(1-\beta \left(v+y^{*}\right)\right)-\left(u+x^{*}\right)\left(v+y^{*}\right), \end{array} \end{array}$$

Application of Taylor series expansion on (u,v)=(0,0) provides the following system:

(8) $$\begin{array}{c} \begin{array}{c} \begin{array}{c} \left[\begin{array}{c} \frac{du}{dt}\\ \frac{dv}{dt} \end{array}\right]=B\left[\begin{array}{c} u\\ v \end{array}\right]+\left[\begin{array}{c} \left(\frac{1}{\eta +y^{*}}\left(\rho -\frac{\rho \;\left(\eta \;y^{*}+{y^{*}}^{2}\right)}{{\left(\eta +y^{*}\right)}^{2}}\right)-\mu \right)vu-\frac{\rho \;x^{*}v^{2}\eta }{{\left(\eta +y^{*}\right)}^{3}}-\frac{\rho \;\eta \;v^{2}u}{{\left(\eta +y^{*}\right)}^{3}}+\frac{\rho \;v^{3}\eta \;x^{*}}{{\left(\eta +y^{*}\right)}^{4}}\\ -\alpha \;v^{2}\beta -uv \end{array}\right] \end{array} \end{array} \end{array}$$

where

$$B=\begin{array}{c} \left[\begin{array}{cc} -\frac{\sigma }{x^{*}} & x^{*}\left(\frac{\eta \rho }{{\left(y^{*}+\eta \right)}^{2}}-\mu \right)\\ -y^{*} & \alpha -x^{*}-2y^{*}\alpha \beta \end{array}\right]. \end{array}$$

Next, we want to convert matrix *B* into its canonical form. For this, the following similarity transformation is considered:

(9) $$\begin{array}{c} \begin{array}{c} \begin{array}{c} \left[\begin{array}{c} u(t)\\ v(t) \end{array}\right]=\left[\begin{array}{cc} x^{*}\left(\frac{\eta \rho }{{\left(y^{*}+\eta \right)}^{2}}-\mu \right) & 0\\ \frac{\sigma }{x^{*}} & \sqrt{x^{*}y^{*}\left(\frac{\eta \rho }{{\left(y^{*}+\eta \right)}^{2}}-\mu \right)+\sigma +\frac{\alpha (2y^{*}\beta -1)\sigma }{x^{*}}} \end{array}\right]\left[\begin{array}{c} w(t)\\ z(t) \end{array}\right] \end{array} \end{array} \end{array}$$

From (8) and (9), it follows that

(10) $$\begin{array}{c} \begin{array}{c} \begin{array}{c} \left[\begin{array}{c} \frac{dw}{dt}\\ \frac{dz}{dt} \end{array}\right]=\left[\begin{array}{cc} 0 & \varphi_{0}\\ -\varphi_{0} & 0 \end{array}\right]\left[\begin{array}{c} w(t)\\ z(t) \end{array}\right] \end{array} \end{array} \end{array}$$

where

$$f\left(w,z\right)=\frac{uv{\left(y^{*}+\eta \right)}^{4}\mu -v\left(v-y^{*}-\eta \right)\eta \left(vx^{*}-u\left(y^{*}+\eta \right)\right)\rho }{x^{*}{\left(y^{*}+\eta \right)}^{4}\mu -x^{*}\eta {\left(y^{*}+\eta \right)}^{2}\rho },$$

and

$$g\left(w,z\right)=\frac{v\left(\frac{\left(x^{*}+\alpha \left(2y^{*}\beta -1\right)\right)\left(u{\left(y^{*}+\eta \right)}^{4}\mu -\eta \left(-v+y^{*}+\eta \right)\left(-vx^{*}+u\left(y^{*}+\eta \right)\right)\rho \right)}{x^{*}{\left(y^{*}+\eta \right)}^{4}\mu -x^{*}\eta {\left(y^{*}+\eta \right)}^{2}\rho }-u-v\alpha \beta \right)}{\sqrt{x^{*}\left(\alpha \left(2-4y^{*}\beta \right)-y^{*}\mu +\frac{y^{*}\eta \rho }{{\left(y^{*}+\eta \right)}^{2}}\right)-{x^{*}}^{2}-\alpha^{2}{\left(1-2y^{*}\beta \right)}^{2}}},$$

$$u=x^{*}\left(\frac{\eta \rho }{{\left(y^{*}+\eta \right)}^{2}}-\mu \right)w,v=\left(\frac{\sigma }{x^{*}}\right)w+\left(\sqrt{x^{*}y^{*}\left(\frac{\eta \rho }{{\left(y^{*}+\eta \right)}^{2}}-\mu \right)+\sigma +\frac{\alpha (2y^{*}\beta -1)\sigma }{x^{*}}}\right)z.$$

Then, the first Lyapunov exponent for system (3) is computed as follows:

$$L\left(\sigma_{0}\right)=L_{1}+L_{2},$$

where

$$\left\{\begin{array}{c} L_{1}=\frac{1}{16}\left(\frac{2\rho y^{*}}{{\left(\eta +y^{*}\right)}^{3}}-\frac{2\rho }{{\left(\eta +y^{*}\right)}^{2}}\right)\\ L_{2}=\frac{-2\alpha \beta -2\alpha \beta \left(\frac{2\rho x^{*}y^{*}}{{\left(\eta +y^{*}\right)}^{3}}-\frac{2\rho x^{*}}{{\left(\eta +y^{*}\right)}^{2}}\right)+\left(\frac{2\rho x^{*}y^{*}}{{\left(\eta +y^{*}\right)}^{3}}-\frac{2\rho x^{*}}{{\left(\eta +y^{*}\right)}^{2}}\right)\left(-\mu -\frac{\rho y^{*}}{{\left(\eta +y^{*}\right)}^{2}}+\frac{\rho }{\eta +y^{*}}\right)}{16\sqrt{-{\left(x^{*}\right)}^{2}-\alpha^{2}{\left(1-2\beta y^{*}\right)}^{2}+x^{*}\left(2\alpha +y^{*}\left(-4\alpha \beta -\mu +\frac{\eta \rho }{{\left(\eta +y^{*}\right)}^{2}}\right)\right)}}. \end{array}\right.$$

Finally, we have the following theorem:

**Theorem** **2.** Assume that conditions (i), (ii), and (iii) are satisfied; then, there exists a unique curve of periodic solutions. Moreover, this curve bifurcates from the fixed point into the region with $\sigma >\sigma_{0}$ if $\left(\rho y^{*}{\left(\eta +y^{*}\right)}^{2}-\rho {\left(\eta +y^{*}\right)}^{3}\right)\sqrt{-{\left(x^{*}\right)}^{2}-\alpha^{2}{\left(1-2\beta y^{*}\right)}^{2}+x^{*}\left(2\alpha +y^{*}\left(-4\alpha \beta -\mu +\frac{\eta \rho }{{\left(\eta +y^{*}\right)}^{2}}\right)\right)}-$ $\alpha \beta {\left(\eta +y^{*}\right)}^{5}+\eta \rho x^{*}\left(\eta \left(2\alpha \beta \eta +\eta \mu -\rho \right)+\left(2\alpha \beta +\mu \right)y^{*}\left(2\eta +y^{*}\right)\right)<0$ or $\left(\rho y^{*}{\left(\eta +y^{*}\right)}^{2}-\rho {\left(\eta +y^{*}\right)}^{3}\right)\sqrt{-{\left(x^{*}\right)}^{2}-\alpha^{2}{\left(1-2\beta y^{*}\right)}^{2}+x^{*}\left(2\alpha +y^{*}\left(-4\alpha \beta -\mu +\frac{\eta \rho }{{\left(\eta +y^{*}\right)}^{2}}\right)\right)}-$ $\alpha \beta {\left(\eta +y^{*}\right)}^{5}+\eta \rho x^{*}\left(\eta \left(2\alpha \beta \eta +\eta \mu -\rho \right)+\left(2\alpha \beta +\mu \right)y^{*}\left(2\eta +y^{*}\right)\right)>0$ if $\sigma <\sigma_{0}.$ In addition, the fixed point is stable whenever $\sigma >\sigma_{0}$ (respectively, $\sigma <\sigma_{0}$) and unstable for $\sigma <\sigma_{0}$ (respectively, $\sigma >\sigma_{0}$) for T<0 and T>0, respectively.

**Figure 3:**
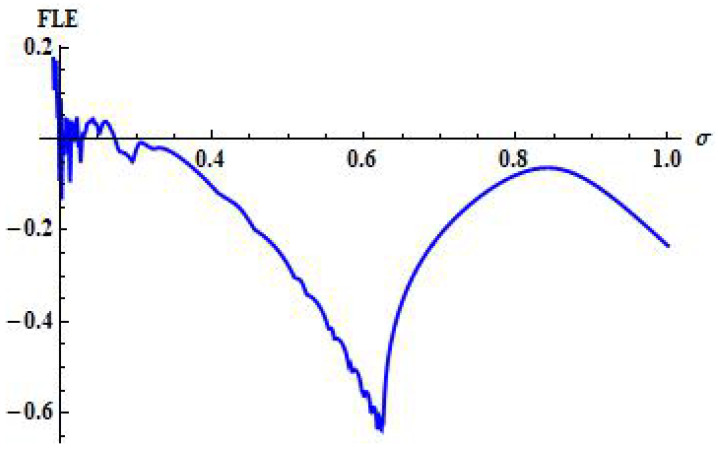
FLE for system (3) for $\alpha =1.636,\;\beta =2\times 10^{-3},\;\delta =0.3743,\;\eta =20.19,\;\mu =0.00311,$ρ=11.131, and σ=0.001181 with initial conditions $x_{0}=1.6197$ and $y_{0}=0.82317$.

The plot of FLE is depicted in Figure 3.

## 3. Boundedness of Solutions

From the second equation of system (5) it follows that

(11) $$y_{n+1}\leq \frac{y_{n}\left(1+h\alpha \right)}{1+h\alpha \beta y_{n}}.$$

Consequently, by solving (11) and then applying the limit, we obtain

(12) $$\lim_{n⟶\infty }supy_{n}\leq \frac{1}{\beta },$$

for all n≥0. In the same way, from the first equation of system (5) we obtain

$$x_{n+1}=\frac{x_{n}+h\left(\sigma +\frac{\rho x_{n}y_{n}}{\eta +y_{n}}\right)}{1+h\left(\delta +\mu y_{n}\right)}$$

(13) $$\leq \frac{x_{n}+h\left(\sigma +\frac{\rho x_{n}}{1+\eta \beta }\right)}{1+h\delta },$$

because

$$x_{n}+h\left(\sigma +\frac{\rho x_{n}y_{n}}{\eta +y_{n}}\right)\leq x_{n}+h\left(\sigma +\frac{\rho x_{n}}{1+\eta \beta }\right)$$

yields

$$y_{n}\leq \frac{1}{\beta }$$

for all n≥0. Then, from (13) we have

$$x_{n+1}\leq \frac{x_{n}\left(1+\frac{h\rho }{1+\eta \beta }\right)+h\sigma }{1+h\delta }.$$

Hence, we can obtain the upper bound for $x_{n}$ as

(14) $$\lim_{n\to \infty }supx_{n}\leq \frac{\sigma +\beta \eta \sigma }{\delta +\beta \delta \eta -\rho },$$

for all n≥0. Finally, we have the following theorem concerning the boundedness of all solutions of (5).

**Theorem** **3.** *Assume that $0<y_{0}\leq \frac{1}{\beta }$ and $0<x_{0}\leq \frac{\sigma +\beta \eta \sigma }{\delta +\beta \delta \eta -\rho }$; then, for all n≥0, every positive solution $\left(x_{n},y_{n}\right)$ of system (5) is bounded and contained in the set $\left[0\;,\;\frac{\sigma +\beta \eta \sigma }{\delta +\beta \delta \eta -\rho }\right]\times \left[0\;,\;\frac{1}{\beta }\right]$ whenever ρ<δ(1+βη).*

## 4. Existence of Fixed Points and Local Stability Analysis

It is easy to see that systems (3) and (5) have more than one fixed point by performing simple algebraic manipulation. Moreover, most of them are complex and have no biological significance. Here, the fixed points with biological significance are boundary fixed points and the unique positive fixed point. In order to obtain those significant fixed points of system (5), we consider the following system of equations:

(15) $$\left\{\begin{array}{c} x^{*}=\frac{x^{*}+h\left(\sigma +\frac{\rho x^{*}y^{*}}{\eta +y^{*}}\right)}{1+h\left(\delta +\mu y^{*}\right)},\\ y^{*}=\frac{y^{*}\left(1+h\alpha \right)}{1+h\left(x^{*}+\alpha \beta y^{*}\right)}. \end{array}\right.$$

Furthermore, from (15) we obtain the following pair:

$$\delta x^{*}+\mu y^{*}=\sigma +\frac{\rho x^{*}y^{*}}{\eta +y^{*}},\;\;y^{*}=\frac{\alpha -x^{*}}{\alpha \beta }.$$

Moreover, we have two fixed points from (15), namely, $\left(\frac{\sigma }{\delta },0\right)$ and $(x^{*},y^{*}),$ where $x^{*}$ is solution of the following equation:

(16) $$a_{11}x^{2}+a_{12}x+a_{13}=0,$$

with

(17) $$\left\{\begin{array}{c} a_{11}=\mu +\alpha \beta \left(\rho -\delta \right),\;\;\;,a_{12}=\alpha \beta \left(\alpha \beta \eta \delta -\eta \mu -\alpha +\sigma +\alpha \rho \right)-2\alpha \mu \\ and\;\;a_{13}=\eta \left(\alpha^{2}\beta \mu -\sigma \right)-\alpha^{2}\left(\beta \sigma -\mu \right). \end{array}\right.$$

In addition, $a_{11},a_{12}>0$ and $a_{13}<0$ if we have

(18) $$\left\{\begin{array}{c} \rho >\delta ,\;\;\;,\sigma +\alpha (\beta \eta \delta +\rho )<\eta \mu +\alpha (1+2\mu )\\ and\;\;\alpha^{2}\mu \left(\beta \eta +1\right)<\sigma \left(\eta +\alpha^{2}\beta \right). \end{array}\right.$$

Hence, using Descartes’ rule of signs (see [41]), we have the following result:

**Theorem** **4.** *Assume that $0<y_{0}\leq \frac{1}{\beta }$ and $0<x_{0}\leq \frac{\sigma +\beta \eta \sigma }{\delta +\beta \delta \eta -\rho }$; then, for*

(19) $$\left\{\begin{array}{c} \rho >\delta ,\;\;\;,\sigma +\alpha (\beta \eta \delta +\rho )<\eta \mu +\alpha (1+2\mu )\\ and\;\;\alpha^{2}\mu \left(\beta \eta +1\right)<\sigma \left(\eta +\alpha^{2}\beta \right) \end{array}\right.$$

there exists a unique positive constant solution $\left(x^{*},y^{*}\right)$ of system (5) in $\left[0\;,\;\frac{\sigma +\beta \eta \sigma }{\delta +\beta \delta \eta -\rho }\right]\times \left[0\;,\;\frac{1}{\beta }\right]$ if and only if $x^{*}<\alpha$ for each $y^{*}\in \left[0,\frac{1}{\beta }\right]$.

In order to discuss the stability of system (5) about these fixed points, we compute the Jacobian matrix $J_{\left(x,y\right)}$ of system (5) about each of its fixed points (x,y). Moreover, $J_{\left(x,y\right)}$ is

$$J_{\left(x,y\right)}=\begin{array}{c} \left[\begin{array}{cc} m_{11} & m_{12}\\ m_{21} & m_{22} \end{array}\right] \end{array}.$$

In addition, the characteristic polynomial C(ϕ) of $J_{\left(x,y\right)}$ is

(20) $$C\left(\phi \right)=\phi^{2}-T\phi +D,$$

where *T* and *D* represent the trace and determinants of $J_{\left(x,y\right)}$, respectively. The following lemma describes the condition equivalent to the Jury conditions for the local stability of fixed points (see [42]).

**Lemma** **1** ([42]). *Let $H\left(\phi \right)=\phi^{2}-T\phi +D$ be the characteristic equation obtained from a 2×2 Jacobian matrix $J_{\left(x,y\right)}.$ Moreover, let $J_{\left(x,y\right)}$ be any Jacobian matrix of system (5) about each of its equilibrium points. Additionally, assume that H(1)>0. Then:*

*(i) $|\phi_{1}|<1$ and $|\phi_{2}|<1$ if and only if H(−1)>0 and D<1*

*(ii) $|\phi_{1}|>1$ and $|\phi_{2}|>1$ if and only if H(−1)>0 and D>1*

*(iii) $|\phi_{1}|<1$ and $|\phi_{2}|>1$ or $(|\phi_{1}|>1$ and $|\phi_{2}<\left|1\right)$ if and only if H(−1)<0*

*(iv) $\phi_{1}$ and $\phi_{2}$ represent complex conjugates with $|\phi_{1}\left|=1=\right|\phi_{2}|$ if and only if $T^{2}-4D<0$ and D=1.*

*As $\phi_{1}$ and $\phi_{2}$ are characteristic values of (20), the point (x,y) is sink if $|\phi_{1}|<1$ and $|\phi_{2}|<1$. Furthermore, it is locally asymptotically stable. The point (x,y) is known as source(repeller) if $|\phi_{1}|>1$ and $|\phi_{2}|>1$. The point (x,y) is a saddle point if $|\phi_{1}|<1$ and $|\phi_{2}|>1$ or $\left(|\phi_{1}\left|>1\;and\;\right|\phi_{2}|<1\right)$. Finally, (x,y) is non-hyperbolic if condition (iv) is satisfied.*

First, we study the stability conditions for (5) about the fixed point $(\frac{\sigma }{\delta },0).$ The matrix $J_{\left(x,y\right)}$ evaluated at $\left(\frac{\sigma }{\delta },0\right)$ is provided by

$$J_{\left(\frac{\sigma }{\delta },0\right)}=\left[\begin{array}{cc} \frac{1}{1+h\delta } & \frac{h(\rho -\eta \mu )\sigma }{\delta (1+h\delta )\eta }\\ 0 & \frac{\delta +h\alpha \delta }{\delta +h\sigma } \end{array}\right].$$

**Figure 4:**
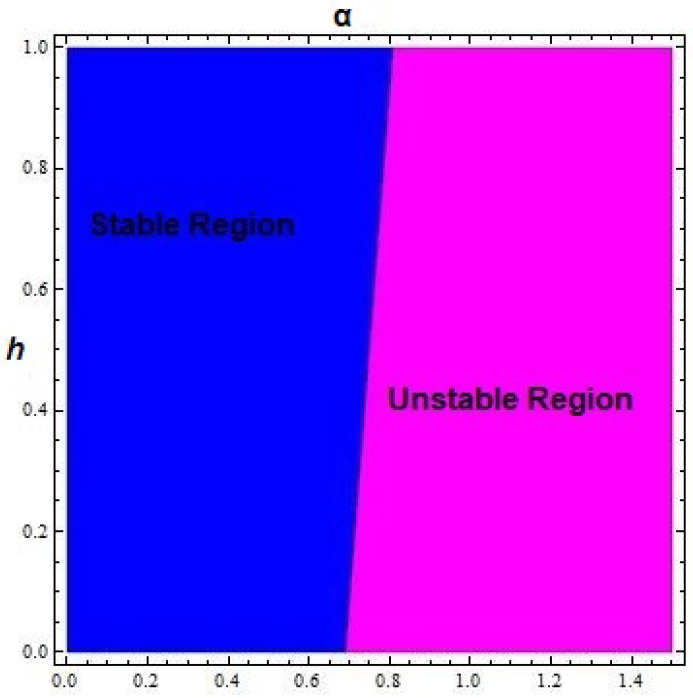
Topological classification of boundary fixed point of system (5) for 0<α<1.5,$\beta =2\times 10^{-3},\;\delta =0.3743,\;\eta =20.19,\;\mu =0.00311,\;\rho =11.131,\;\sigma =0.001181$ and 0<h<1 with initial conditions $x_{0}=1.6197$, and $y_{0}=8.2317$.

Furthermore, the eigenvalues of $J_{\left(\frac{\sigma }{\delta },0\right)}$ are $\xi_{1}=\frac{1}{1+h\delta }$ and $\xi_{2}=\frac{\delta +h\alpha \delta }{\delta +h\sigma }$ with $|\xi_{1}|<1$ for all parametric values. Hence, we have the following result related to the dynamics of (5) about $\left(\frac{\sigma }{\delta },0\right)$:

**Proposition** **1.** *The boundary equilibrium $\left(\frac{\sigma }{\delta },0\right)$ of system (5) is source and saddle iff conditions αδ<σ and αδ>σ are satisfied respectively (see Figure 4).*

Next, our task is to explore of the local stability of system (5) about the point $\left(x^{*},y^{*}\right)$. Let $J_{\left(x^{*},y^{*}\right)}$ be the Jacobian matrix of system (5) about the fixed point $\left(x^{*},y^{*}\right)$; then, $J_{\left(x^{*},y^{*}\right)}$ has the following mathematical form:

$$J_{\left(x^{*},y^{*}\right)}=\left[\begin{array}{cc} \frac{1+\frac{h\rho y^{*}}{\eta +y^{*}}}{1+h\delta +h\mu y^{*}} & -\frac{h\left(h\mu \sigma {\left(\eta +y^{*}\right)}^{2}+x^{*}\left(\eta \left(\eta \mu -\left(1+h\delta \right)\rho \right)+\mu y^{*}\left(2\eta +y^{*}+h\rho y^{*}\right)\right)\right)}{{\left(\eta +y^{*}\right)}^{2}{\left(1+h\delta +h\mu y^{*}\right)}^{2}}\\ -\frac{hy^{*}}{1+h\alpha } & 1-\frac{h\alpha \beta y^{*}}{1+h\alpha } \end{array}\right].$$

Moreover, from $J_{\left(x^{*},y^{*}\right)}$ we can obtain the following characteristic polynomial:

(21) $$\begin{array}{ccc} C(\phi ) & = & \phi^{2}-\phi \left(1-\frac{h\alpha \beta y^{*}}{1+h\alpha }+\frac{1+\frac{h\rho y^{*}}{\eta +y^{*}}}{1+h\delta +h\mu y^{*}}\right)+D, \end{array}$$

where

$$\begin{array}{ccc} D & = & \frac{1}{1+h\delta +h\mu y^{*}}+\frac{hy^{*}}{1+h\alpha }\left(\frac{S{\left(1+h\alpha \right)}^{2}}{{\left(1+hx^{*}+h\alpha \beta y^{*}\right)}^{2}}-\frac{\alpha \beta }{1+h\delta +h\mu y^{*}}+\frac{\rho +h\alpha \rho -h\alpha \beta \rho y^{*}}{\left(\eta +y^{*}\right)\left(1+h\delta +h\mu y^{*}\right)}\right) \end{array}$$

and

$$\begin{array}{c} S=-\frac{h\left(h\mu \sigma {\left(\eta +y^{*}\right)}^{2}+x^{*}\left(\eta \left(\eta \mu -\left(1+h\delta \right)\rho \right)+\mu y^{*}\left(2\eta +y^{*}+h\rho y^{*}\right)\right)\right)}{{\left(\eta +y^{*}\right)}^{2}{\left(1+h\delta +h\mu y^{*}\right)}^{2}}. \end{array}$$

By considering (19) and taking

(22) $$\left\{\begin{array}{c} s_{1}=h\left(h\mu \sigma {\left(\eta +y^{*}\right)}^{2}+x^{*}\left(\eta \left(\eta \mu -\left(1+h\delta \right)\rho \right)+\mu y^{*}\left(2\eta +y^{*}+h\rho y^{*}\right)\right)\right),\\ s_{2}={\left(\eta +y^{*}\right)}^{2}{\left(1+h\delta +h\mu y^{*}\right)}^{2}, \end{array}\right.$$

it follows that

$$C\left(1\right)=\frac{hy^{*}\left(\left(S+h\left(S+\alpha \beta \right)\delta \right)\eta +y^{*}\left(S\left(1+h\delta +h\eta \mu \right)+h\alpha \beta \left(\delta +\eta \mu -\rho \right)+h\left(S+\alpha \beta \right)\mu y^{*}\right)\right)}{\left(1+h\alpha \right)\left(\eta +y^{*}\right)\left(1+h\delta +h\mu y^{*}\right)}>0,$$

for

$$hs_{2}\alpha \beta \left(\delta \left(1+\eta \right)+\mu \left(\eta +y^{*}\right)\right)>s_{1}\left(\eta \left(1+h\delta \right)+y^{*}\left(1+h\left(\delta +2\mu \eta \right)\right)\right)+hs_{2}\alpha \beta \rho .$$

Moreover, we have

(23) $$\begin{array}{c} h=\frac{\delta \eta +y^{*}\left(\delta +\eta \left(\alpha \beta +\mu -S\right)-\rho +\left(\alpha \beta +\mu -S\right)y^{*}\right)}{y^{*}\left(S\delta \eta -\alpha \left(\delta +\eta \mu -\rho \right)+y^{*}\left(S\left(\delta +\eta \mu \right)-\alpha \left(\mu +\beta \rho \right)+S\mu y^{*}\right)\right)-\alpha \delta \eta } \end{array}$$

and

$$\begin{array}{ccc} C(-1) & = & 1+\frac{\left(1+h\alpha \right)\left(1+hx^{*}\right)}{{\left(1+hx^{*}+h\alpha \beta y^{*}\right)}^{2}}+\frac{1+\frac{h\rho y^{*}}{\eta +y^{*}}}{1+h\delta +h\mu y^{*}}+\frac{1}{1+h\delta +h\mu y^{*}}\\ & + & \frac{hy^{*}}{1+h\alpha }\left(\frac{S{\left(1+h\alpha \right)}^{2}}{{\left(1+hx^{*}+h\alpha \beta y^{*}\right)}^{2}}-\frac{\alpha \beta }{1+h\delta +h\mu y^{*}}+\frac{\rho +h\alpha \rho -h\alpha \beta \rho y^{*}}{\left(\eta +y^{*}\right)\left(1+h\delta +h\mu y^{*}\right)}\right). \end{array}$$

Hence, for the study of the linearized stability of system (5) about $(x^{*},y^{*}),$ we have the following proposition (see Lemma 1).

**Proposition** **2.** Let (18) remain true; then, $\left(x^{*},y^{*}\right)$ is positive equilibrium of (5). Moreover, let us assume that

(24) $$\left\{\begin{array}{c} \Omega_{11}=1+hx^{*}+h\alpha \beta y^{*},\;\;\Omega_{12}=1+h\delta +h\mu y^{*},\;\;\Omega_{13}=\eta +y^{*},\\ \Omega_{14}=1+h\alpha , \end{array}\right.$$

*then,*

- The fixed point $\left(x^{*},y^{*}\right)$ is stable if and only if for
$$\begin{array}{c} \eta x^{*}\left(\rho +2\mu y^{*}\right)<h\mu \sigma x^{*}{\left(y^{*}\right)}^{2}+\mu \left(h\sigma +x^{*}\right){\left(\eta +y^{*}\right)}^{2} \end{array}$$
  *we have*
$$\begin{array}{c} \Omega_{11}^{2}\Omega_{12}\Omega_{13}>\Omega_{14}\left(\Omega_{11}\left(\Omega_{13}+h\rho y^{*}\right)+hy^{*}\left(\delta \eta +\left(hS\delta -\alpha \beta \right)\Omega_{13}+hS\eta \mu y^{*}-\alpha \beta \rho y^{*}\right)\right) \end{array}$$
- The fixed point $\left(x^{*},y^{*}\right)$ is non-hyperbolic if and only if
$$h=\frac{\delta \eta +y^{*}\left(\delta +\eta \left(\alpha \beta +\mu -S\right)-\rho +\left(\alpha \beta +\mu -S\right)y^{*}\right)}{y^{*}\left(S\delta \eta -\alpha \left(\delta +\eta \mu -\rho \right)+y^{*}\left(S\left(\delta +\eta \mu \right)-\alpha \left(\mu +\beta \rho \right)+S\mu y^{*}\right)\right)-\alpha \delta \eta }$$
  *and*
(25) $$\begin{array}{c} 1+\frac{\Omega_{13}+h\rho y^{*}}{\Omega_{13}\Omega_{12}}<\frac{h\alpha \beta y^{*}}{\Omega_{14}}+4\left(\frac{1}{\Omega_{12}}+\frac{hy^{*}}{\Omega_{14}}\left(\frac{S\Omega_{14}^{2}}{{\left(\Omega_{11}\right)}^{2}}-\frac{\alpha \beta }{\Omega_{12}}+\frac{\rho +h\alpha \rho -h\alpha \beta \rho y^{*}}{\left(\eta +y^{*}\right)\left(\Omega_{12}\right)}\right)\right). \end{array}$$

**Remark** **1.** *Let (18) remain true; then, there is no chance of system (5) undergoing period-doubling bifurcation, as C(−1)>0 for every σ,ρ,η,δ,μ,α,β>0 and ρ>δ.*

We now have the following theorem for the possible validation of Remark 1.

**Theorem** **5.** Let (18) remain true and let ρ>δ. Moreover, let $\left(x^{*},y^{*}\right)$ be the positive fixed point of system (5) and

$$\begin{array}{ccc} C(-1) & = & 1+\frac{\left(1+h\alpha \right)\left(1+hx^{*}\right)}{{\left(1+hx^{*}+h\alpha \beta y^{*}\right)}^{2}}+\frac{1+\frac{h\rho y^{*}}{\eta +y^{*}}}{1+h\delta +h\mu y^{*}}+\frac{1}{1+h\delta +h\mu y^{*}}\\ & + & \frac{hy^{*}}{1+h\alpha }\left(\frac{S{\left(1+h\alpha \right)}^{2}}{{\left(1+hx^{*}+h\alpha \beta y^{*}\right)}^{2}}-\frac{\alpha \beta }{1+h\delta +h\mu y^{*}}+\frac{\rho +h\alpha \rho -h\alpha \beta \rho y^{*}}{\left(\eta +y^{*}\right)\left(1+h\delta +h\mu y^{*}\right)}\right). \end{array}$$

*Then, C(−1)>0 for every σ,ρ,η,δ,μ,α,β>0.*

**Proof.**  Assume (18) and ρ>δ. Additionally, let $\left(x^{*},y^{*}\right)$ be the positive fixed point of system (5) and

$$\begin{array}{ccc} C(-1) & = & 1+\frac{\left(1+h\alpha \right)\left(1+hx^{*}\right)}{{\left(1+hx^{*}+h\alpha \beta y^{*}\right)}^{2}}+\frac{1+\frac{h\rho y^{*}}{\eta +y^{*}}}{1+h\delta +h\mu y^{*}}+\frac{1}{1+h\delta +h\mu y^{*}}\\ & + & \frac{hy^{*}}{1+h\alpha }\left(\frac{S{\left(1+h\alpha \right)}^{2}}{{\left(1+hx^{*}+h\alpha \beta y^{*}\right)}^{2}}-\frac{\alpha \beta }{1+h\delta +h\mu y^{*}}+\frac{\rho +h\alpha \rho -h\alpha \beta \rho y^{*}}{\left(\eta +y^{*}\right)\left(1+h\delta +h\mu y^{*}\right)}\right). \end{array}$$

Then, from

$$\begin{array}{c} S=-\frac{h\left(h\mu \sigma {\left(\eta +y^{*}\right)}^{2}+x^{*}\left(\eta \left(\eta \mu -\left(1+h\delta \right)\rho \right)+\mu y^{*}\left(2\eta +y^{*}+h\rho y^{*}\right)\right)\right)}{{\left(\eta +y^{*}\right)}^{2}{\left(1+h\delta +h\mu y^{*}\right)}^{2}}, \end{array}$$

we have

$$\begin{array}{c} h\mu \sigma {\left(\eta +y^{*}\right)}^{2}+x^{*}\left(\eta \left(\eta \mu -\left(1+h\delta \right)\rho \right)+\mu y^{*}\left(2\eta +y^{*}+h\rho y^{*}\right)\right)>0 \end{array}$$

$$\begin{array}{c} \Leftrightarrow h\mu \sigma y^{*}\left(\eta +x^{*}y^{*}\right)+\mu \left(h\sigma +x^{*}\right)\left(\eta^{2}+\eta y^{*}+{\left(y^{*}\right)}^{2}\right)>\eta x^{*}\left(\rho +\mu y^{*}\right) \end{array}$$

(26) $$\begin{array}{c} \Leftrightarrow \eta x^{*}\left(\rho +2\mu y^{*}\right)<h\mu \sigma x^{*}{\left(y^{*}\right)}^{2}+\mu \left(h\sigma +x^{*}\right){\left(\eta +y^{*}\right)}^{2}. \end{array}$$

Finally, under condition (26), we have C(−1)>0 if and only if

$$\begin{array}{c} \left(\eta +y^{*}\right){\left(1+hx^{*}+h\alpha \beta y^{*}\right)}^{2}\left(1+h\delta +h\mu y^{*}\right)> \end{array}$$

$$\begin{array}{c} \left(1+h\alpha \right)\left(\eta +y^{*}+h\left(x^{*}\left(\eta +y^{*}+h\rho y^{*}\right)+y^{*}\left(S\left(\eta +h\delta \eta \right)+\rho +Sy^{*}\left(1+h\delta +h\eta \mu +h\mu y^{*}\right)\right)\right)\right) \end{array}$$

$$\begin{array}{c} \Leftrightarrow \Omega_{11}^{2}\Omega_{12}\Omega_{13}>\Omega_{14}\left(h\delta \eta x^{*}+\Omega_{13}\left(1+hx^{*}\right)+hy^{*}\left(hS\eta \left(\delta +\mu \right)+\rho +h\rho x^{*}+S\Omega_{12}y^{*}\right)\right) \end{array}$$

(27) $$\begin{array}{c} \Leftrightarrow \Omega_{11}^{2}\Omega_{12}\Omega_{13}>\Omega_{14}\left(\Omega_{11}\left(\Omega_{13}+h\rho y^{*}\right)+hy^{*}\left(\delta \eta +\left(hS\delta -\alpha \beta \right)\Omega_{13}+hS\eta \mu y^{*}-\alpha \beta \rho y^{*}\right)\right). \end{array}$$

where, $\Omega_{11},\Omega_{12},\Omega_{13},\Omega_{14}$ are defined in (24). Hence, under condition (26), the above inequality (27) is true for every choice of σ,ρ,η,δ,μ,α,β>0 and ρ>δ. This completes the proof. □

## 5. Neimark–Sacker Bifurcation

This section is related to the bifurcation analysis of system (5) about $(x_{*},y_{*}).$ Moreover, all conditions for the existence and positivity of $\left(x_{*},y_{*}\right)$ are provided in Section 2. The Neimark–Sacker bifurcation in discrete-time mathematical systems corresponds to the Hopf bifurcation in continuous-time systems. For example, when Neimark–Sacker bifurcation is supercritical, a stable centre loses its stability. A parameter, namely, the bifurcation parameter, is varied with the resulting birth of an established quasi-cycle or cycle. Moreover, we mention any of these as an invariant closed curve. Additionally, for subcritical Neimark–Sacker bifurcation, a stable centre bounded by an unstable closed arc loses its stability through a resulting vanishing of the invariant closed curve as a bifurcation parameter is varied. Here, we discuss the Neimark–Sacker bifurcation experienced by system (5) about $\left(x_{*},y_{*}\right)$ under certain mathematical conditions. For further study of bifurcation theory and to better understand this surprising behaviuor of discrete-time mathematical systems, we refer readers to [42,43,44,45,46,47,48]. Here, we use the standard theory of bifurcation for study of the Neimark–Sacker bifurcation of system (5) at $\left(x_{*},y_{*}\right)$. Assume that

$$\begin{array}{c} S=-\frac{h\left(h\mu \sigma {\left(\eta +y^{*}\right)}^{2}+x^{*}\left(\eta \left(\eta \mu -\left(1+h\delta \right)\rho \right)+\mu y^{*}\left(2\eta +y^{*}+h\rho y^{*}\right)\right)\right)}{{\left(\eta +y^{*}\right)}^{2}{\left(1+h\delta +h\mu y^{*}\right)}^{2}}, \end{array}$$

then, one can see from Proposition 2 that roots $\phi_{1},\;\phi_{2}$ of (21) are complex and satisfy $|\phi_{1}\left|=\right|\phi_{2}|=1$ if and only if

$$\begin{array}{c} h=\frac{\delta \eta +y^{*}\left(\delta +\eta \left(-S+\alpha \beta +\mu \right)-\rho +\left(-S+\alpha \beta +\mu \right)y^{*}\right)}{-\alpha \delta \eta +y^{*}\left(S\delta \eta -\alpha \left(\delta +\eta \mu -\rho \right)+y^{*}\left(S\left(\delta +\eta \mu \right)-\alpha \left(\mu +\beta \rho \right)+S\mu y^{*}\right)\right)} \end{array}$$

and

$$\begin{array}{c} 1-\frac{h\alpha \beta y^{*}}{1+h\alpha }+\frac{1+\frac{h\rho y^{*}}{\eta +y^{*}}}{1+h\delta +h\mu y^{*}}<4D, \end{array}$$

where

$$\begin{array}{ccc} D & = & \frac{\eta +h\alpha \eta +y^{*}\left(1+h(\alpha +S\eta -\alpha \beta \eta +hS\delta \eta +\rho +h\alpha \rho )\right)}{\left(1+h\alpha \right)\left(\eta +y^{*}\right)\left(1+h\delta +h\mu y^{*}\right)}\\ & + & \frac{y^{*}\left(hy^{*}\left(S\left(1+h\delta +h\eta \mu \right)-\alpha \beta \left(1+h\rho \right)+hS\mu y^{*}\right)\right)}{\left(1+h\alpha \right)\left(\eta +y^{*}\right)\left(1+h\delta +h\mu y^{*}\right)}. \end{array}$$

Furthermore, under the suppositions that

(28) $$\left\{\begin{array}{c} \rho >\delta ,\;\;\;,\sigma +\alpha (\beta \eta \delta +\rho )<\eta \mu +\alpha (1+2\mu )\\ and\;\;\alpha^{2}\mu \left(\beta \eta +1\right)<\sigma \left(\eta +\alpha^{2}\beta \right) \end{array}\right.$$

we study the following set:

$$\Psi_{*}=\left\{\sigma ,\rho ,\eta ,\delta ,\mu ,\alpha ,\beta \in {ℜ}^{+},h=\frac{\delta \eta +y^{*}\left(\delta +\eta \left(-S+\alpha \beta +\mu \right)-\rho +\left(-S+\alpha \beta +\mu \right)y^{*}\right)}{-\alpha \delta \eta +y^{*}\left(S\delta \eta -\alpha \left(\delta +\eta \mu -\rho \right)+y^{*}\left(S\left(\delta +\eta \mu \right)-\alpha \left(\mu +\beta \rho \right)+S\mu y^{*}\right)\right)}\right\}.$$

Then, the positive fixed point $\bar{X}$ of system (5) experiences Neimark–Sacker bifurcation such that *h* is taken as the bifurcation parameter and varies slightly in the neighborhood of $\hat{h},$ which is provided as

$$\begin{array}{c} \hat{h}=\frac{\delta \eta +y^{*}\left(\delta +\eta \left(-S+\alpha \beta +\mu \right)-\rho +\left(-S+\alpha \beta +\mu \right)y^{*}\right)}{-\alpha \delta \eta +y^{*}\left(S\delta \eta -\alpha \left(\delta +\eta \mu -\rho \right)+y^{*}\left(S\left(\delta +\eta \mu \right)-\alpha \left(\mu +\beta \rho \right)+S\mu y^{*}\right)\right)}. \end{array}$$

In addition, assume that $\left(\sigma ,\rho ,\eta ,\delta ,\mu ,\alpha ,\beta \right)\in \Psi_{2}$; then, system (5) is characterized equivalently with the following planar map:

(29) $$\left(\begin{array}{c} x\\ y \end{array}\right)\to \left(\begin{array}{c} \frac{x+\hat{h}\left(\sigma +\frac{\rho xy}{\eta +y}\right)}{1+\hat{h}\left(\delta +\mu y\right)}\\ \frac{y\left(1+\hat{h}\alpha \right)}{1+\hat{h}\left(x+\alpha \beta y\right)} \end{array}\right).$$

In order to discuss and analyze the normal form theory for Neimark–Sacker bifurcation for fixed point $\bar{X}=\left(x^{*},y^{*}\right)$ of (29), we suppose that $h_{1}$ represents a small perturbation in $\hat{h}$. Then, the perturbed mapping for (29) can be described by the next map:

(30) $$\left(\begin{array}{c} x\\ y \end{array}\right)\to \left(\begin{array}{c} \frac{x+\left(\hat{h}+h_{1}\right)\left(\sigma +\frac{\rho xy}{\eta +y}\right)}{1+\left(\hat{h}+h_{1}\right)\left(\delta +\mu y\right)}\\ \frac{y\left(1+(\hat{h}+h_{1})\alpha \right)}{1+\left(\hat{h}+h_{1}\right)\left(x+\alpha \beta y\right)} \end{array}\right).$$

By taking $\bar{p}=x-x^{*},$$\bar{z}=y-x^{*}$ and $h=\hat{h}+h_{1},$ from (30) we can obtain the following mapping with an equilibrium point at (0,0):

(31) $$\left(\begin{array}{c} \bar{p}\\ \bar{z} \end{array}\right)\to M\left(\begin{array}{c} \bar{p}\\ \bar{z} \end{array}\right)+\left(\begin{array}{c} F_{1}\left(\bar{p},\bar{z}\right)\\ F_{2}\left(\bar{p},\bar{z}\right) \end{array}\right),$$

where

(32) $$\begin{array}{c} M=\left(\begin{array}{cc} \frac{1+\frac{hy\rho }{y+\eta }}{1+h(\delta +y\mu )} & \frac{h\left(-\frac{xy\rho }{{\left(y+\eta \right)}^{2}}+\frac{x\rho }{y+\eta }\right)}{1+h(\delta +y\mu )}-\frac{h\mu \left(x+h\left(\frac{xy\rho }{y+\eta }+\sigma \right)\right)}{{\left(1+h\left(\delta +y\mu \right)\right)}^{2}}\\ -\frac{hy(1+h\alpha )}{{\left(1+h\left(x+y\alpha \beta \right)\right)}^{2}} & \frac{\left(1+hx)(1+h\alpha \right)}{{\left(1+h\left(x+y\alpha \beta \right)\right)}^{2}} \end{array}\right), \end{array}$$

and

$$\begin{array}{ccc} F_{1}\left(\bar{p},\bar{z}\right) & = & \left(\frac{-h{\left(y^{*}+\eta \right)}^{2}\mu +h\left(\eta +h\delta \eta -h{y^{*}}^{2}\mu \right)\rho }{{\left(y^{*}+\eta \right)}^{2}{\left(1+h\left(\delta +x^{*}\mu \right)\right)}^{2}}\right)\bar{p}\bar{z}\\ & + & \left(\frac{h\left(\frac{2x^{*}y^{*}\rho }{{\left(y^{*}+\eta \right)}^{3}}-\frac{2x^{*}\rho }{{\left(y^{*}+\eta \right)}^{2}}\right)}{2(1+h\left(\delta +y^{*}\mu \right))}-\frac{h^{2}\mu \left(-\frac{x^{*}y^{*}\rho }{{\left(y^{*}+\eta \right)}^{2}}+\frac{x^{*}\rho }{y^{*}+\eta }\right)}{{\left(1+h\left(\delta +y^{*}\mu \right)\right)}^{2}}+\frac{h^{2}\mu^{2}\left(x^{*}+h\left(\frac{x^{*}y^{*}\rho }{x^{*}+\eta }+\sigma \right)\right)}{{\left(1+h\left(\delta +y^{*}\mu \right)\right)}^{3}}\right){\bar{z}}^{2}\\ & + & \left(\frac{\frac{2hy^{*}\rho }{{\left(y^{*}+\eta \right)}^{3}}-\frac{2h\rho }{{\left(y^{*}+\eta \right)}^{2}}}{2(1+h\left(\delta +y^{*}\mu \right))}-\frac{h\mu \left(-\frac{hy^{*}\rho }{{\left(y^{*}+\eta \right)}^{2}}+\frac{h\rho }{y^{*}+\eta }\right)}{{\left(1+h\left(\delta +y^{*}\mu \right)\right)}^{2}}+\frac{h^{2}\mu^{2}\left(1+\frac{hy^{*}\rho }{y^{*}+\eta }\right)}{{\left(1+h\left(\delta +y^{*}\mu \right)\right)}^{3}}\right)\bar{p}{\bar{z}}^{2}\\ & + & \left(\frac{h\left(-\frac{6x^{*}y^{*}\rho }{{\left(y^{*}+\eta \right)}^{4}}+\frac{6x^{*}\rho }{{\left(y^{*}+\eta \right)}^{3}}\right)}{6(1+h\left(\delta +y^{*}\mu \right))}-\frac{h^{2}\mu \left(\frac{2x^{*}y^{*}\rho }{{\left(y^{*}+\eta \right)}^{3}}-\frac{2x^{*}\rho }{{\left(y^{*}+\eta \right)}^{2}}\right)}{2{\left(1+h\left(\delta +y^{*}\mu \right)\right)}^{2}}+\frac{h^{3}\mu^{2}\left(-\frac{x^{*}y^{*}\rho }{{\left(y+\eta \right)}^{2}}+\frac{x^{*}\rho }{y^{*}+\eta }\right)}{{\left(1+h\left(\delta +y^{*}\mu \right)\right)}^{3}}\right){\bar{z}}^{3}\\ & - & \left(\frac{h^{3}\mu^{3}\left(x^{*}+h\left(\frac{x^{*}y^{*}\rho }{y^{*}+\eta }+\sigma \right)\right)}{{\left(1+h\left(\delta +y^{*}\mu \right)\right)}^{4}}\right){\bar{z}}^{3}, \end{array}$$

$$\begin{array}{ccc} F_{2}\left(\bar{p},\bar{z}\right) & = & \left(\frac{h^{2}y^{*}\left(1+h\alpha \right)}{{\left(1+hx^{*}+hy^{*}\alpha \beta \right)}^{3}}\right){\bar{p}}^{2}+\left(\frac{2h^{2}y^{*}\alpha \left(1+h\alpha \right)\beta }{{\left(1+h\left(x^{*}+y^{*}\alpha \beta \right)\right)}^{3}}-\frac{h(1+h\alpha )}{{\left(1+h\left(x^{*}+y^{*}\alpha \beta \right)\right)}^{2}}\right)\bar{p}\bar{z}\\ & - & \left(\frac{h\left(1+hx^{*}\right)\alpha \left(1+h\alpha \right)\beta }{{\left(1+h\left(x^{*}+y^{*}\alpha \beta \right)\right)}^{3}}\right){\bar{z}}^{2}-\left(-\frac{h^{3}y^{*}\left(1+h\alpha \right)}{{\left(1+h\left(x^{*}+y^{*}\alpha \beta \right)\right)}^{4}}\right){\bar{p}}^{3}\\ & + & \left(\frac{h^{2}\left(1+h\alpha \right)\left(1+h\left(x^{*}-2y^{*}\alpha \beta \right)\right)}{{\left(1+h\left(x^{*}+y^{*}\alpha \beta \right)\right)}^{4}}\right){\bar{p}}^{2}\bar{z}+\left(\frac{h^{2}\alpha \left(1+h\alpha \right)\beta \left(2+2hx^{*}-hy^{*}\alpha \beta \right)}{{\left(1+h\left(x^{*}+y^{*}\alpha \beta \right)\right)}^{4}}\right)\bar{p}{\bar{z}}^{2}\\ & + & \left(\frac{h^{2}\left(1+hx^{*}\right)\alpha^{2}\left(1+h\alpha \right)\beta^{2}}{{\left(1+h\left(x^{*}+y^{*}\alpha \beta \right)\right)}^{4}}\right){\bar{z}}^{3}. \end{array}$$

The characteristic equation S(ϕ)=0 generated by the Jacobian matrix of (31) about (0,0) can be written as

(33) $$\phi^{2}-\hat{T}\left(h_{1}\right)\phi +\hat{D}\left(h_{1}\right)=0,$$

where

$$\begin{array}{ccc} \hat{D}\left(h_{1}\right) & = & \frac{\eta +\left(\hat{h}+h_{1}\right)\alpha \eta +y^{*}\left(1+\left(\hat{h}+h_{1}\right)\left(\alpha +S\eta -\alpha \beta \eta +\left(\hat{h}+h_{1}\right)S\delta \eta +\rho +\left(\hat{h}+h_{1}\right)\alpha \rho \right)\right)}{\left(1+\left(\hat{h}+h_{1}\right)\alpha \right)\left(\eta +y^{*}\right)\left(1+\left(\hat{h}+h_{1}\right)\delta +\left(\hat{h}+h_{1}\right)\mu y^{*}\right)}\\ & + & \frac{y^{*}\left(\left(\hat{h}+h_{1}\right)y^{*}\left(S\left(1+\left(\hat{h}+h_{1}\right)\delta +\left(\hat{h}+h_{1}\right)\eta \mu \right)-\alpha \beta \left(1+\left(\hat{h}+h_{1}\right)\rho \right)+\left(\hat{h}+h_{1}\right)S\mu y^{*}\right)\right)}{\left(1+\left(\hat{h}+h_{1}\right)\alpha \right)\left(\eta +y^{*}\right)\left(1+\left(\hat{h}+h_{1}\right)\delta +\left(\hat{h}+h_{1}\right)\mu y^{*}\right)}, \end{array}$$

$$\begin{array}{ccc} \hat{T}\left(h_{1}\right) & = & 1-\frac{\left(\hat{h}+h_{1}\right)\alpha \beta y^{*}}{1+(\hat{h}+h_{1})\alpha }+\frac{1+\frac{\left(\hat{h}+h_{1}\right)\rho y^{*}}{\eta +y^{*}}}{1+\left(\hat{h}+h_{1}\right)\delta +\left(\hat{h}+h_{1}\right)\mu y^{*}}. \end{array}$$

Consider that $\left(\sigma ,\rho ,\eta ,\delta ,\mu ,\alpha ,\beta \right)\in \Psi_{2}$; at that point, the complex solutions for (33) are calculated as follows:

$$\begin{array}{c} \phi_{1}=\frac{\hat{T}\left(h_{1}\right)-i\sqrt{4\hat{D}\left(h_{1}\right)-{\hat{T}}^{2}\left(h_{1}\right)}}{2} \end{array}$$

and

$$\begin{array}{c} \phi_{2}=\frac{\hat{T}\left(h_{1}\right)+i\sqrt{4\hat{D}\left(h_{1}\right)-{\hat{T}}^{2}\left(h_{1}\right)}}{2}. \end{array}$$

Moreover, we have

(34) $${\left(\frac{d|\phi_{1}|}{dh_{1}}\right)}_{h_{1}=0}\neq 0$$

because $|\hat{T}\left(0\right)|<2$ as $\left(\sigma ,\rho ,\eta ,\delta ,\mu ,\alpha ,\beta \right)\in \Psi_{2}.$ Moreover, a simple computation yields that

$$\begin{array}{ccc} \hat{T}\left(0\right) & = & 1-\frac{\hat{h}\alpha \beta y^{*}}{1+\hat{h}\alpha }+\frac{1+\frac{\hat{h}\rho y^{*}}{\eta +y^{*}}}{1+\hat{h}\delta +\hat{h}\mu y^{*}}, \end{array}$$

and we suppose that $\hat{T}\left(0\right)\neq 0$ and $\hat{T}\left(0\right)\neq 1$, that is,

(35) $$\begin{array}{c} 1+\frac{1+\frac{\hat{h}\rho y^{*}}{\eta +y^{*}}}{1+\hat{h}\delta +\hat{h}\mu y^{*}}\neq \frac{\hat{h}\alpha \beta y^{*}}{1+\hat{h}\alpha },\;\;\;\;\frac{1+\frac{\hat{h}\rho y^{*}}{\eta +y^{*}}}{1+\hat{h}\delta +\hat{h}\mu y^{*}}\neq \frac{\hat{h}\alpha \beta y^{*}}{1+\hat{h}\alpha }. \end{array}$$

Suppose that (35) holds and $\left(\sigma ,\rho ,\eta ,\delta ,\mu ,\alpha ,\beta \right)\in \Psi_{2}.$ Then, it follows that $\hat{T}\left(0\right)\neq \pm 2,0,-1$, that is, $\phi_{1}^{m},\phi_{2}^{m}\neq 1$ for every m∈{1,2,3,4} about $h_{1}=0$. Consequently, both solutions of (33) do not lie inside the intersection of the unit circle with the coordinate axes when $h_{1}=0.$ In the same way, we assume that $\lambda =\frac{\hat{T}\left(0\right)}{2}$, $\omega =\frac{1}{2}\sqrt{4\hat{D}\left(0\right)-{\hat{T}}^{2}\left(0\right)}.$ Formerly, to change (31) into normal form, we used the following similarity transformation:

(36) $$\left(\begin{array}{c} \bar{p}\\ \bar{z} \end{array}\right)=\left(\begin{array}{cc} l_{12} & 0\\ \lambda -l_{11} & -\omega \end{array}\right)\left(\begin{array}{c} u\\ v \end{array}\right).$$

By using (36), we obtain the next typical form for (31):

(37) $$\left(\begin{array}{c} u\\ v \end{array}\right)\to \left(\begin{array}{cc} \lambda & -\omega \\ \omega & \lambda \end{array}\right)\left(\begin{array}{c} u\\ v \end{array}\right)+\left(\begin{array}{c} \tilde{F}\left(u,v\right)\\ \tilde{G}\left(u,v\right) \end{array}\right).$$

Moreover, we have

$$\begin{array}{ccc} \tilde{F}\left(u,v\right) & = & \left({\left(\left(\lambda -l_{11}\right)u-\omega \;v\right)}^{2}l_{15}+\left(\left(\lambda -l_{11}\right)u-\omega \;v\right)l_{13}\right)u\\ & + & \frac{{\left(\left(\lambda -l_{11}\right)u-\omega \;v\right)}^{3}l_{16}+{\left(\left(\lambda -l_{11}\right)u-\omega \;v\right)}^{2}l_{14}}{l_{12}}+O\left(\left(|u\right|+{\left|v|\right)}^{4}\right), \end{array}$$

$$\begin{array}{ccc} \tilde{G}\left(u,v\right) & = & \left(\frac{\left(\lambda -l_{11}\right)\left({\left(\left(\lambda -l_{11}\right)u-\omega \;v\right)}^{2}l_{15}+\left(\left(\lambda -l_{11}\right)u-\omega \;v\right)l_{13}\right)}{l_{12}\omega }\right)l_{12}u-\frac{l_{26}{l_{12}}^{3}u^{3}}{\omega }\\ & - & \frac{\left(\left(\lambda -l_{11}\right)u-\omega \;v\right)l_{27}{l_{12}}^{2}u^{2}}{\omega }-\left(\frac{{\left(\left(\lambda -l_{11}\right)u-\omega \;v\right)}^{2}l_{28}+\left(\left(\lambda -l_{11}\right)u-\omega \;v\right)l_{24}}{\omega }\right)l_{12}u\\ & + & \frac{\left(\lambda -l_{11}\right)\left({\left(\left(\lambda -l_{11}\right)u-\omega \;v\right)}^{3}l_{16}+{\left(\left(\lambda -l_{11}\right)u-\omega \;v\right)}^{2}l_{14}\right)}{l_{12}\omega }\\ & - & \frac{{\left(\left(\lambda -l_{11}\right)u-\omega \;v\right)}^{3}l_{29}+l_{12}^{2}u^{2}l_{23}+{\left(\left(\lambda -l_{11}\right)u-\omega \;v\right)}^{2}l_{25}}{\omega }+O\left(\left(|u\right|+{\left|v|\right)}^{4}\right). \end{array}$$

where $l_{11},l_{12},l_{13}$ and $l_{14}$ are respective elements of M. In addition, $l_{1j}$ for j=3,4,5,6 and $l_{2j}$ for j=3,4,…,9 are coefficients from expressions $F_{1}\left(\bar{p},\bar{z}\right)$ and $F_{2}\left(\bar{p},\bar{z}\right)$, respectively. Now, we describe the next non-zero real numbers:

$$ϝ={\left(\left[-Re\left(\frac{\left(1-2\phi_{1}\right)\phi_{2}^{2}}{1-\phi_{1}}\xi_{20}\xi_{11}\right)-\frac{1}{2}|\xi_{11}{|}^{2}-{\left|\xi_{02}\right|}^{2}+Re\left(\phi_{2}\xi_{21}\right)\right]\right)}_{h_{1}=0},$$

where

$$\begin{array}{ccc} & \xi_{20}= & \frac{1}{8}\left(2\left(\lambda -l_{11}\right)l_{13}-\frac{2\omega^{2}l_{14}}{l_{12}}+\frac{2\left(\lambda -l_{11}\right){}^{2}l_{14}}{l_{12}}\right)\\ & + & \frac{1}{4}\left(-\frac{2\left(\lambda -l_{11}\right){}^{2}l_{14}}{l_{12}}+l_{12}\left(-\frac{\left(\lambda -l_{11}\right)l_{13}}{l_{12}}+l_{24}\right)+2\left(\lambda -l_{11}\right)l_{25}\right)\\ & + & \frac{i}{4}\left(-\frac{\omega \left(\lambda -l_{11}\right)l_{14}}{l_{12}}+\frac{\left(\lambda -l_{11}\right){}^{3}l_{14}}{\omega l_{12}}-\left(-\omega l_{13}-\frac{2\omega \left(\lambda -l_{11}\right)l_{14}}{l_{12}}\right)\right)\\ & + & l_{12}\frac{i}{4}\left(\left(\frac{\left(\lambda -l_{11}\right){}^{2}l_{13}}{\omega l_{12}}-\frac{\left(\lambda -l_{11}\right)l_{24}}{\omega }\right)+\omega l_{25}-\frac{l_{12}^{2}l_{23}+\left(\lambda -l_{11}\right){}^{2}l_{25}}{\omega }\right) \end{array}$$

$$\begin{array}{ccc} & \xi_{11}= & \frac{1}{2}\left(\left(\lambda -l_{11}\right)l_{13}+\frac{\omega^{2}l_{14}}{l_{12}}+\frac{\left(\lambda -l_{11}\right){}^{2}l_{14}}{l_{12}}+i\left(\frac{\omega \left(\lambda -l_{11}\right)l_{14}}{l_{12}}+\frac{\left(\lambda -l_{11}\right){}^{3}l_{14}}{\omega l_{12}}\right)\right)\\ & + & \frac{i}{2}\left(l_{12}\left(\frac{\left(\lambda -l_{11}\right){}^{2}l_{13}}{\omega l_{12}}-\frac{\left(\lambda -l_{11}\right)l_{24}}{\omega }\right)-\omega l_{25}-\frac{l_{12}^{2}l_{23}+\left(\lambda -l_{11}\right){}^{2}l_{25}}{\omega }\right), \end{array}$$

$$\begin{array}{ccc} & \xi_{02}= & \frac{1}{4}\left(\left(\lambda -l_{11}\right)l_{13}-\frac{\omega^{2}l_{14}}{l_{12}}+\frac{\left(\lambda -l_{11}\right){}^{2}l_{14}}{l_{12}}+\frac{2\left(\lambda -l_{11}\right){}^{2}l_{14}}{l_{12}}+l_{12}\left(\frac{\left(\lambda -l_{11}\right)l_{13}}{l_{12}}-l_{24}\right)\right)\\ & + & \frac{1}{4}\left(\left(2\left(\lambda -l_{11}\right)l_{25}\right)+i\left(-\frac{\omega \left(\lambda -l_{11}\right)l_{14}}{l_{12}}+\frac{\left(\lambda -l_{11}\right){}^{3}l_{14}}{\omega l_{12}}+\omega l_{25}-\omega l_{13}\right)\right)\\ & - & \frac{i}{4}\left(\frac{2\omega \left(\lambda -l_{11}\right)l_{14}}{l_{12}}-l_{12}\left(\frac{\left(\lambda -l_{11}\right){}^{2}l_{13}}{\omega l_{12}}-\frac{\left(\lambda -l_{11}\right)l_{24}}{\omega }\right)+\frac{l_{12}^{2}l_{23}+\left(\lambda -l_{11}\right){}^{2}l_{25}}{\omega }\right), \end{array}$$

and

$$\begin{array}{ccc} & \xi_{21}= & \frac{1}{8}\left(\omega^{2}l_{15}+3\left(\lambda -l_{11}\right){}^{2}l_{15}+l_{12}^{2}l_{27}-l_{12}\left(\frac{2\left(\lambda -l_{11}\right){}^{2}l_{15}}{l_{12}}-2\left(\lambda -l_{11}\right)l_{28}\right)\right)\\ & + & \frac{1}{8}\left(3\omega^{2}l_{29}+3\left(\lambda -l_{11}\right){}^{2}l_{29}+i\left(2\omega \left(\lambda -l_{11}\right)l_{15}+\frac{3\omega^{3}l_{16}}{l_{12}}+\frac{6\omega \left(\lambda -l_{11}\right){}^{2}l_{16}}{l_{12}}\right)\right)\\ & + & \frac{i}{8}\left(\frac{3\left(\lambda -l_{11}\right){}^{4}l_{16}}{\omega l_{12}}-\frac{3l_{12}^{3}l_{26}}{\omega }-\frac{3\left(\lambda -l_{11}\right)l_{12}^{2}l_{27}}{\omega }+l_{12}\left(\frac{\omega \left(\lambda -l_{11}\right)l_{15}}{l_{12}}-\omega l_{28}\right)\right)\\ & + & \frac{i}{8}\left(3l_{12}\left(\frac{\left(\lambda -l_{11}\right){}^{3}l_{15}}{\omega l_{12}}-\frac{\left(\lambda -l_{11}\right){}^{2}l_{28}}{\omega }\right)-3\omega \left(\lambda -l_{11}\right)l_{29}-\frac{3\left(\lambda -l_{11}\right){}^{3}l_{29}}{\omega }\right). \end{array}$$

Hence, we have the following theorem.

**Theorem** **6.** *Assume that (35) holds true and ϝ≠0. Then, the positive fixed point*

$$\bar{X}=\left(x^{*},y^{*}\right)$$

*of system (5) experiences Neimark–Sacker bifurcation whenever h changes in the least neighborhood of*

$$\begin{array}{c} \hat{h}=\frac{\delta \eta +y^{*}\left(\delta +\eta \left(-S+\alpha \beta +\mu \right)-\rho +\left(-S+\alpha \beta +\mu \right)y^{*}\right)}{-\alpha \delta \eta +y^{*}\left(S\delta \eta -\alpha \left(\delta +\eta \mu -\rho \right)+y^{*}\left(S\left(\delta +\eta \mu \right)-\alpha \left(\mu +\beta \rho \right)+S\mu y^{*}\right)\right)}. \end{array}$$

*In addition, if ϝ<0,(ϝ>0), respectively, then an attracting or repelling invariant closed curve bifurcates from the equilibrium point for $h>\hat{h}\left(h<\hat{h}\right)$, respectively.*

## 6. Control of Neimark–Sacker Bifurcation

The study of chaos theory and bifurcation control is a multidisciplinary area of mathematics research that concentrates on basic designs and extremely complex categorical laws of primary conditions in any dynamical systems that are supposed to have entirely arbitrary statuses of disorder and inconsistency. Generally, the leading standard of disorder defines how a minor variation in any state of a nonlinear dynamical system can result in significant changes in an advanced state (the implication being that a complex dependency on primary conditions is close) [48]. Every disordered attractor encloses a countless amount of periodic and unstable orbits. Chaotic behaviour at any time is a gesture where the state system moves in the vicinity of any of these regions for a time and then drops to a closer periodic and unstable orbit, where it hangs for a degree of time, etcetera [48]. Chaos control stabilizes any of these irregular periodic orbits by the worth of small structure perturbations. Hence, we use a simple chaos control method for system (5). Furthermore, many well-known techniques have been developed in previous decades to control chaos in any discrete dynamical system. We refer readers to [48,49,50] for additional details connected to these methods. Here, we implement a generalized hybrid control technique to control the Neimark–Sacker bifurcation (seecite [51,52,53]). The generalized hybrid control method [48] is centred on parameter perturbation and a state feedback control technique. By applying generalized hybrid control methodology (with control parameter b∈(0,1]) to system (5), we obtain

(38) $$\left\{\begin{array}{c} x_{n+1}=\sin b\left(\frac{x_{n}+h\left(\sigma +\frac{\rho x_{n}y_{n}}{\eta +y_{n}}\right)}{1+h\left(\delta +\mu y_{n}\right)}\right)+\left(1-\sin b\right)x_{n},\\ y_{n+1}=\sin b\left(\frac{y_{n}\left(1+h\alpha \right)}{1+h\left(x_{n}+\alpha \beta y_{n}\right)}\right)+\left(1-\sin b\right)y_{n}. \end{array}\right.$$

Then, system (38) is controllable provided that its fixed point $\left(x^{*},y^{*}\right)$ is locally asymptotically stable. Additionally, the Jacobian matrix for system (38) at its positive fixed point $\left(x^{*},y^{*}\right)$ is calculated as follows:

(39) $$J_{c}=\left[\begin{array}{cc} 1-\sin b+\frac{\sin b\left(1+\frac{h\rho y^{*}}{\eta +y^{*}}\right)}{1+h\delta +h\mu y^{*}} & -\frac{h\sin b\left(h\mu \sigma {\left(\eta +y^{*}\right)}^{2}+x^{*}\left(\eta \left(\eta \mu -\left(1+h\delta \right)\rho \right)+\mu y^{*}\left(2\eta +y^{*}+h\rho y^{*}\right)\right)\right)}{{\left(\eta +y^{*}\right)}^{2}{\left(1+h\delta +h\mu y^{*}\right)}^{2}}\\ -\frac{h\sin b\left(1+h\alpha \right)y^{*}}{{\left(1+hx^{*}+h\alpha \beta y^{*}\right)}^{2}} & 1-\sin b+\frac{\sin b\left(1+h\alpha \right)\left(1+hx^{*}\right)}{{\left(1+hx^{*}+h\alpha \beta y^{*}\right)}^{2}} \end{array}\right]$$

where

(40) $$Trace\left[J_{c}\right]=2-2\sin b+\frac{\sin b\left(1+h\alpha \right)\left(1+hx^{*}\right)}{{\left(1+hx^{*}+h\alpha \beta y^{*}\right)}^{2}}+\frac{\sin b\left(1+\frac{h\rho y^{*}}{\eta +y^{*}}\right)}{1+h\delta +h\mu y^{*}}$$

and

(41) $$\left\{\begin{array}{c} Det\left[J_{c}\right]=\frac{\left(1-h\tilde{A}\delta -h\tilde{A}\mu y^{*}\right)\left(1+h\sin b\alpha -h\tilde{A}\left(x^{*}+\alpha \beta y^{*}\right)\left(2+hx^{*}+h\alpha \beta y^{*}\right)\right)}{{\left(1+hx^{*}+h\alpha \beta y^{*}\right)}^{2}\left(1+h\delta +h\mu y^{*}\right)}\\ -\frac{h\sin b\left(1+h\alpha \right)x^{*}\left(\left(1-h\tilde{A}\delta \right)\eta +y^{*}\left(1-h\tilde{A}\left(\delta +\eta \mu \right)+h\sin b\rho -h\tilde{A}\mu y^{*}\right)\right)}{\left(\eta +y^{*}\right){\left(1+hx^{*}+h\alpha \beta y^{*}\right)}^{2}\left(1+h\delta +h\mu y^{*}\right)}\\ +h\sin by^{*}\left(\frac{S(\sin b+h\sin b\alpha )}{{\left(1+hx^{*}+h\alpha \beta y^{*}\right)}^{2}}+\frac{\rho \left(1+\sin b\left(\frac{1+h\alpha }{{\left(1+hx^{*}+h\alpha \beta y^{*}\right)}^{2}}-1\right)\right)}{\left(\eta +y^{*}\right)\left(1+h\delta +h\mu y^{*}\right)}\right), \end{array}\right.$$

with $\sin b-1=\tilde{A}.$ Moreover, we have the following result:

**Theorem** **7.** Assume the fixed point $\left(x^{*},y^{*}\right)$ of system (38). Then, $\left(x^{*},y^{*}\right)$ is locally asymptotically stable ⟺ we have

$$\begin{array}{c} \left|Trace[J_{c}]\right|<1+Det\left[J_{c}\right]<2, \end{array}$$

*where $Trace[J_{c}]$ and $Det[J_{c}]$ are as defined in (40) and (41), respectively.*

## 7. Numerical Simulations

**Figure 5:**
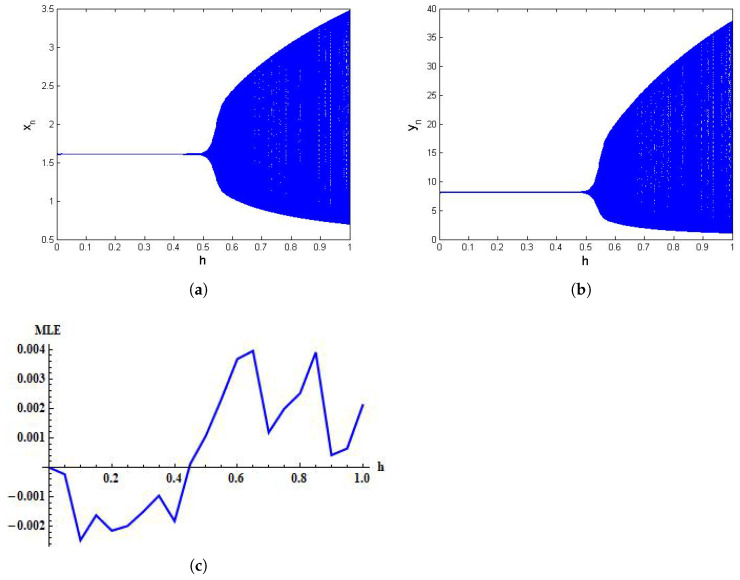
Bifurcation diagrams for system (5) for $\alpha =1.636,\;\beta =2\times 10^{-3},\;\delta =0.3743,\;\eta =20.19,$μ=0.00311,ρ=l.131,σ=0.1181, and h∈[0,1) with initial conditions $x_{0}=1.6197$ and $y_{0}=8.2317$. (**a**) Bifurcation diagram for $x_{n}$; (**b**) Bifurcation diagram for $y_{n}$; (**c**) MLE.

First, assume that $\alpha =1.636,\;\beta =2\times 10^{-3},\;\delta =0.3743,\;\eta =20.19,\;\mu =0.00311,$ρ=1.131,σ=0.1181, and h∈[0,1). Then, from system (5) we have $\left(x^{*},y^{*}\right)=(1.61954,$ 8.2317). Moreover, in this case $x_{0}=1.61954$ and $y_{0}=8.2317$ are initial conditions. The graphical behavior of both variables is shown in Figure 5. It can be seen that $x_{n}$ and $y_{n}$ undergo Neimark–Sacker bifurcation at unique positive fixed points $\left(x^{*},y^{*}\right)=\left(1.61954,8.2317\right).$ In addition, in Figure 5c the maximum Lyapunov exponents are represented. To understand the consistency between bifurcation diagrams and Lyapunov exponents, in Figure 5a, one can see that bifurcation in the first variable occurs when *h* passes through h=0.5, and the Lyapunov exponent changes from negative to positive as *h* crosses the horizontal line at h=0.5.

**Figure 6:**
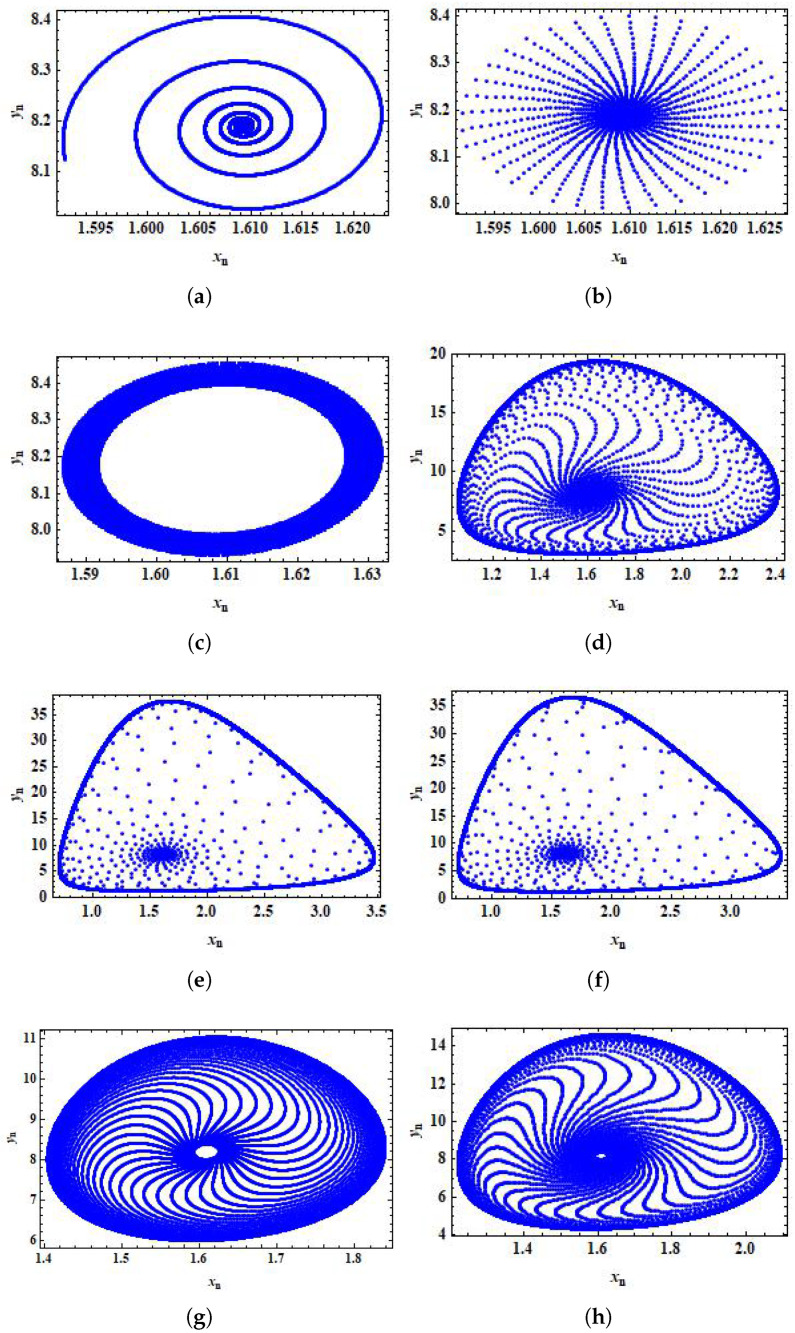
Phase portraits for system (5) for $\alpha =1.636,\;\beta =2\times 10^{-3},\;\delta =0.3743,\;\eta =20.19,$μ=0.00311,ρ=1.131,σ=0.1181, and h∈[0,1) with initial conditions $x_{0}=1.6197$ and $y_{0}=8.2317$. (**a**) Phase portrait for h=0.014912; (**b**) phase portrait for h=0.40891412; (**c**) phase portrait for h=0.491289; (**d**) phase portrait for h=0.591289; (**e**) phase portrait for h=0.991289; (**f**) phase portrait for h=0.961289; (**g**) phase portrait for h=0.521289; (**h**) phase portrait for h=0.531289.

In this case, we have ϝ=−0.00065841<0, which verifies Theorem 6. Moreover, we have C(−1)=3.2136789>0, which validates Theorem 5. By varying the stepsize, *h*, in [0,1) phase portraits for system (5) can be obtained, as shown in Figure 6. Hence, we can observe that system (5) experiences Neimark–Sacker bifurcation when the parameter *h* certainly passes through h=0.4917267952 (see Figure 6c). Second, to discuss the feasibility of the designed control technique, we take $\alpha =1.636,\;\beta =2\times 10^{-3},\;\delta =0.3743,\;\eta =20.19,$μ=0.00311,ρ=1.131,σ=0.1181 and h∈[0,1).

**Figure 7:**
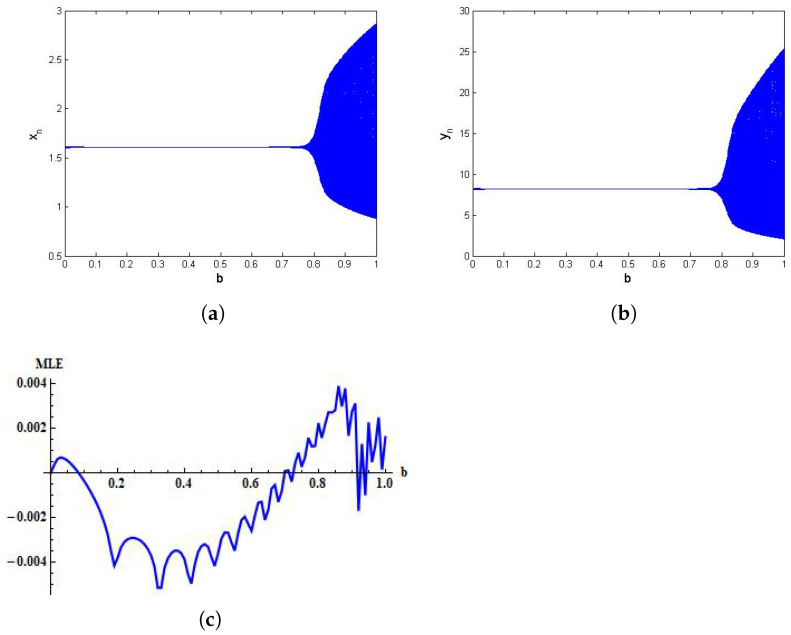
Controlled plots of system (38) for $\alpha =1.636,\;\beta =2\times 10^{-3},\;\delta =0.3743,\;\eta =20.19,$μ=0.00311,ρ=1.131,σ=0.1181,h∈[0,1), and b∈[0,1) with initial conditions $x_{0}=1.6197$ and $y_{0}=8.2317$. (**a**) Bifurcation diagram for $x_{n}$; (**b**) Bifurcation diagram for $y_{n}$; (**c**) MLE.

Then, from system (38), we have $\left(x^{*},y^{*}\right)=\left(1.61954,8.2317\right).$ Moreover, by taking *b* as the control parameter, it can be observed from Figure 7 that the Neimark–Sacker bifurcation at unique positive fixed point $\left(x^{*},y^{*}\right)$ is effectively controlled for a large range of the control parameter b. Additionally, the MLE for controlled system (38) is provided in Figure 7c. From Figure 7a, it can be seen that bifurcation in the first variable occurs when *b* lies between 0<b<0.1. Moreover, the controlled system is stable in 0.1<b<0.69, and the Lyapunov exponent changes from positive to negative at the point b=0.1(approx) and negative to positive as *b* crosses the horizontal line at b=0.69, which shows the consistency of controlled plots with corresponding MLE.

**Figure 8:**
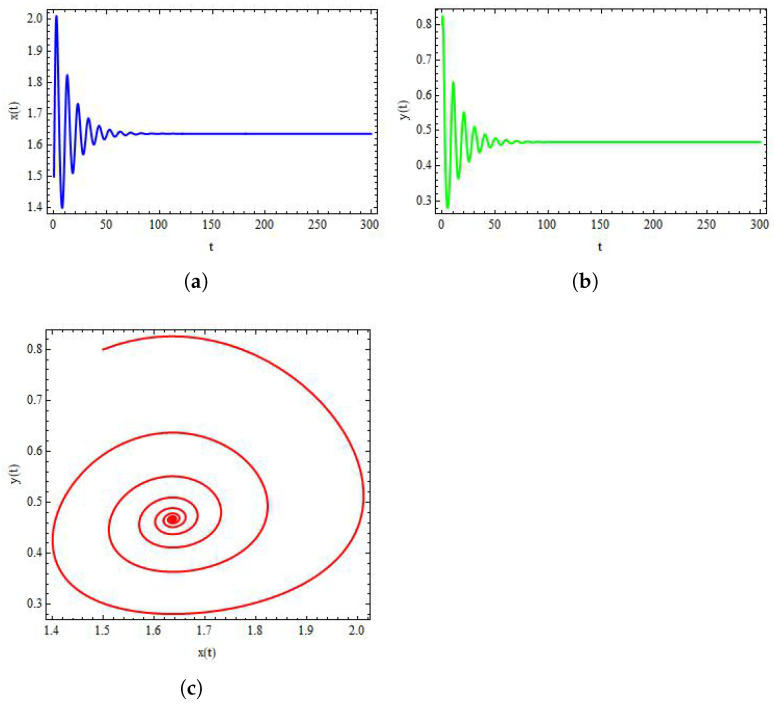
Plots of system (3) for $\alpha =1.636,\;\beta =2\times 10^{-3},\;\delta =0.3743,\;\eta =20.19,\;\mu =0.00311,$ρ=11.131, and σ=0.2181 with initial conditions $x_{0}=1.6197$, and $y_{0}=0.82317$. (**a**) Plot of x(t) for system (3); (**b**) plot of y(t) for system (3); (**c**) stable phase portrait for system (3).

**Figure 9:**
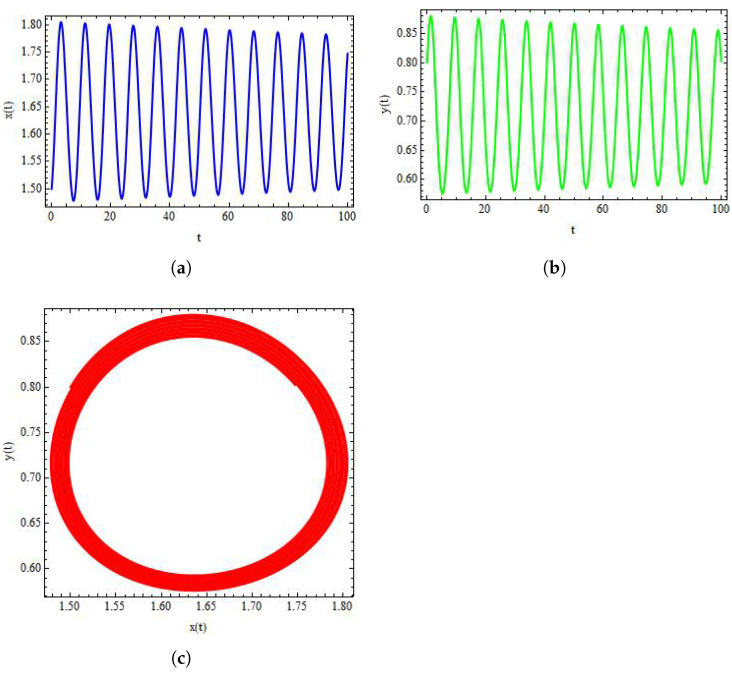
Plots of system (3) for $\alpha =1.636,\;\beta =2\times 10^{-3},\;\delta =0.3743,\;\eta =20.19,\;\mu =0.00311,$ρ=11.131, and σ=0.001181 with initial conditions $x_{0}=1.6197$ and $y_{0}=0.82317$. (**a**) Plot of x(t) for system (3); (**b**) plot of y(t) for system (3); (**c**) phase portrait for system (3).

Finally, to discuss the dynamics of system (3), we take $\alpha =1.636,\;\beta =2\times 10^{-3},$δ=0.3743,η=20.19,μ=0.00311,ρ=11.131, and σ=0.1181. Then, from system (3) we have $\left(x^{*},y^{*}\right)=\left(1.519,0.823\right).$ Consequently, we obtain a stable system (3) for these values. In addition, the qualitative behaviour of system (3) is shown in Figure 8. On the other side, by taking σ as bifurcation parameter and taking σ=0.001181, it can be observed from Figure 9 that the system (3) experiences Hopf bifurcation at positive fixed point $(x^{*},y^{*}).$ Additionally, the plots of both variables for system (3) are provided in Figure Figure 9a,b. In this case, the value of FLE is calculated as approximately $L(\sigma_{0})=-0.00645890013.$

## 8. Conclusions

Here, we have considered an immunogenic tumor model for discretization and qualitative study. The original model (3) was presented and studied by Kuznetsov et al. [28] in its continuous form. Moreover, they studied the dynamics of (3) in its continuous form as well. This work is focused on the study of the consistent counterpart of (3) and comparing the dynamics of model (3) with its discrete-time counterpart, which has not been performed previously for this model. Hence, we first converted the system (3) into its discrete form using a consistency-preserving discretization method. For this purpose, a nonstandard finite difference method was applied to obtain a discrete counterpart of the particular model (3). Our examination exposes that the continuous system (3) undergoes Hopf bifurcation about its positive fixed point σ, which is considered a bifurcation parameter, and passes over a critical value $\sigma_{0}=x^{*}\left(\alpha -x^{*}-2y^{*}\alpha \beta \right)$, such as ρ>δ. Furthermore, the first Lyapunov exponent is calculated in the closed form, specified as follows:

$$L\left(\sigma_{0}\right)=L_{1}+L_{2},$$

where

$$\left\{\begin{array}{c} L_{1}=\frac{1}{16}\left(\frac{2\rho y^{*}}{{\left(\eta +y^{*}\right)}^{3}}-\frac{2\rho }{{\left(\eta +y^{*}\right)}^{2}}\right)\\ L_{2}=\frac{-2\alpha \beta -2\alpha \beta \left(\frac{2\rho x^{*}y^{*}}{{\left(\eta +y^{*}\right)}^{3}}-\frac{2\rho x^{*}}{{\left(\eta +y^{*}\right)}^{2}}\right)+\left(\frac{2\rho x^{*}y^{*}}{{\left(\eta +y^{*}\right)}^{3}}-\frac{2\rho x^{*}}{{\left(\eta +y^{*}\right)}^{2}}\right)\left(-\mu -\frac{\rho y^{*}}{{\left(\eta +y^{*}\right)}^{2}}+\frac{\rho }{\eta +y^{*}}\right)}{16\sqrt{-{\left(x^{*}\right)}^{2}-\alpha^{2}{\left(1-2\beta y^{*}\right)}^{2}+x^{*}\left(2\alpha +y^{*}\left(-4\alpha \beta -\mu +\frac{\eta \rho }{{\left(\eta +y^{*}\right)}^{2}}\right)\right)}}. \end{array}\right.$$

On the other hand, when *h* is taken as a bifurcation parameter, the discrete-time version, which is obtained using a nonstandard finite difference scheme, experiences Neimark–Sacker bifurcation about its positive fixed point $\left(x^{*},y^{*}\right)$ whenever *h* passes through

$$\begin{array}{c} \hat{h}=\frac{\delta \eta +y^{*}\left(\delta +\eta \left(-S+\alpha \beta +\mu \right)-\rho +\left(-S+\alpha \beta +\mu \right)y^{*}\right)}{-\alpha \delta \eta +y^{*}\left(S\delta \eta -\alpha \left(\delta +\eta \mu -\rho \right)+y^{*}\left(S\left(\delta +\eta \mu \right)-\alpha \left(\mu +\beta \rho \right)+S\mu y^{*}\right)\right)}. \end{array}$$

The conditions for the presence of Neimark–Sacker bifurcation are specified in Theorem 6. Mathematical simulation exposes that our discretization is bifurcation-conserving and equal with lesser step size value; the first Lyapunov exponents are approximately the same in both cases, that is, $ϝ\approx L(\sigma_{0})=-0.006832.$ From Theorem 5, it can be seen that there is no chance of period-doubling bifurcation in our discrete-time system, which shows the consistency of the discretizing technique used here. Moreover, to analyze the wide-ranging and rich dynamics of another discrete-time counterpart of the immunogenic tumors model, it is possible to use the Euler method or piecewise constant arguments with system (3). Using the Euler method or piecewise constant arguments, it is possible to discuss other types of bifurcations and chaos control. We refer readers to [44,45,46,47,48] and the references therein for further consideration. We anticipate that analysis of system (3) using the Euler method and bifurcation analysis with chaos control for the obtained system will be our future tasks.

## Figures and Tables

**Table 1:** Parameter estimates from [28].

System Parameters	Dimensional Parameters	Estimated Values
σ	$\sigma =\frac{s}{nE_{0}T_{0}}$	0.001181
ρ	$\rho =\frac{p}{nT_{0}}$	11.131
η	$\eta =\frac{g}{T_{0}}$	20.19
μ	$\mu =\frac{m}{n}$	0.00311
δ	$\delta =\frac{d}{nT_{0}}$	0.3743
α	$\alpha =\frac{a}{nT_{0}}$	1.636
β	$\beta =bnT_{0}$	$2.0\times 10^{-3}$

## Data Availability

Not applicable.
